# Relationships between Circulating Urea Concentrations and Endometrial Function in Postpartum Dairy Cows

**DOI:** 10.3390/ani5030382

**Published:** 2015-08-14

**Authors:** Zhangrui Cheng, Chike F. Oguejiofor, Theerawat Swangchan-Uthai, Susan Carr, D. Claire Wathes

**Affiliations:** Department of Production and Population Health, Royal Veterinary College, North Mymms, Hertfordshire AL9 7TA, UK; E-Mails: zcheng@rvc.ac.uk (Z.C.); coguejiofor@rvc.ac.uk (C.F.O.); theerawat.s@chula.ac.th (T.S.-U.); scarr@rvc.ac.uk (S.C.)

**Keywords:** protein metabolism, innate immunity, endometrium, involution, cholesterol

## Abstract

**Simple Summary:**

Dairy cows fed high levels of protein to increase milk yield tend to have reduced fertility but the reasons behind this are unclear. Differing dietary protein levels are reflected in altered urea concentrations in both blood and other tissues including the uterus. We showed that the circulating urea concentration was highly correlated to changed expression levels of many genes in the endometrium shortly after calving. These were predominantly associated with tissue repair, innate immunity and lipid metabolism. A subsequent study found no effect of altered urea concentration on endometrial gene expression *in vitro* implying that the dietary influence is indirect.

**Abstract:**

Both high and low circulating urea concentrations, a product of protein metabolism, are associated with decreased fertility in dairy cows through poorly defined mechanisms. The rate of involution and the endometrial ability to mount an adequate innate immune response after calving are both critical for subsequent fertility. Study 1 used microarray analysis to identify genes whose endometrial expression 2 weeks postpartum correlated significantly with the mean plasma urea per cow, ranging from 3.2 to 6.6 mmol/L. The biological functions of 781 mapped genes were analysed using Ingenuity Pathway Analysis. These were predominantly associated with tissue turnover (e.g., *BRINP1*, *FOXG1*), immune function (e.g., *IL17RB*, *CRISPLD2*), inflammation (e.g., *C3*, *SERPINF1*, *SERPINF2*) and lipid metabolism (e.g., *SCAP*, *ACBD5*, *SLC10A*). Study 2 investigated the relationship between urea concentration and expression of 6 candidate genes (*S100A8*, *HSP5A*, *IGF1R*, *IL17RB*, *BRINP1*, *CRISPLD2*) in bovine endometrial cell culture. These were treated with 0, 2.5, 5.0 or 7.5 mmol/L urea, equivalent to low, medium and high circulating values with or without challenge by bacterial lipopolysaccharide (LPS). LPS increased *S100A8* expression as expected but urea treatment had no effect on expression of any tested gene. Examination of the genes/pathways involved suggests that plasma urea levels may reflect variations in lipid metabolism. Our results suggest that it is the effects of lipid metabolism rather than the urea concentration which probably alter the rate of involution and innate immune response, in turn influencing subsequent fertility.

## 1. Introduction

Nutrition plays a crucial role in the reproductive physiology and endocrinology of the dairy cow. Following parturition, the lactating cow undergoes an increased demand for nutrients such as glucose, amino acids and fatty acids required for milk production [1,2]. This demand typically exceeds the dietary intake of the high-yielding dairy cow hence body energy reserves are mobilised, resulting in a state of negative energy balance (NEB) which may last for several weeks postpartum [3,4]. A conflict in metabolic needs arise in which modern dairy cows may prioritize milk production to the detriment of reproductive functions and fertility [2,5,6]. During NEB, circulating concentrations of many metabolites and metabolic hormones (non esterified fatty acids (NEFA), beta-hydroxybutyrate (BHB), insulin, glucose, insulin-like growth factor 1 (IGF-1)) are altered and these influence fertility directly by altering ovarian, follicular or uterine functions, or indirectly by modulating the somatotrophic/gonadotrophic axis [2,6]. The extent to which cows enter NEB in the immediate postpartum period is strongly predictive of their fertility later in that lactation [5,6].

Many dairy cows are fed high protein diets in order to maximise milk production [7]. Urea is a product of protein breakdown and the peripheral urea nitrogen concentration reflects protein metabolism in dairy cattle [8]. Urea diffuses into body fluids such as blood and milk and equilibrates in other parts of the body, including the reproductive tissues [9]. Blood urea concentrations often fluctuate around calving [10] and are influenced by a wide variety of interrelated parameters including dietary protein intake and body requirement and metabolism [11]. The main cause of high circulating urea is an excess intake of total N including rumen degradable protein [12,13]. Energy deficit may also stimulate catabolism of amino acids from tissue proteins leading to increased urea production [1]. In addition, impaired liver function during NEB reduces the metabolic clearance of urea [14].

Both high and low circulating urea concentrations have been associated with reduced fertility in dairy cows, in particular an increased calving to conception interval [7,10,15,16]. These results are however inconsistent between trials and the link(s) between fertility and protein metabolism have yet to be established conclusively. Urea itself is potentially toxic but Laven *et al*. [12] concluded that much of the deleterious effect of increased intakes of degradable protein was probably mediated by post prandial increases in ammonia which can have harmful effects on both oocytes and early embryos [17,18]. Another proposed mechanism has been that excess dietary protein decreases uterine pH or alters other aspects of the uterine environment during the early luteal phase thus making it less favourable for embryo survival [19,20,21,22].

The bovine uterus must undergo extensive remodelling after calving in order to restore normal tissue architecture after expulsion of the calf and placenta. This involves a major reduction in size, necrosis of the surface endometrium and extensive restructuring of the extracellular matrix [23,24]. Tissue debris accumulates in the uterine lumen contributing to a lochial discharge. Following an initial period of degradation, tissue repair is initiated and the caruncles remodel and regenerate epithelium. This process is superficially complete by 3–4 weeks postpartum, but the deeper layers are not fully restored until 6–8 weeks [25]. We have previously provided evidence that many components of the IGF system are expressed in the involuting uterus and that IGF1 is likely to be a key growth factor contributing to the rate of repair [26,27]. In most dairy cows the uterus also acquires bacterial contamination at calving. Although the infection is normally cleared within 2–3 weeks, about 15% of animals develop a persistent endometritis associated with inflammation [28,29]. This condition causes reduced fertility and is associated with longer intervals to conception [30].

This paper describes two experiments designed to test the hypothesis that factors influencing circulating urea concentrations during the immediate postpartum period modify endometrial function and that this in turn may contribute to the observed associations between urea and fertility. In Study 1, we utilized results of an experiment in which two groups of dairy cows were managed by differential feeding and milking to produce mild or severe NEB in the early postpartum period [31]. Correlation analysis was performed between the mean plasma urea concentration postpartum and the normalized mRNA expression intensity of individual genes within the endometrium, harvested 2 weeks after calving during the period of involution when a robust innate immune response is crucial to overcome infection. In order to validate these findings, Study 2 investigated the effect of urea concentration on endometrial gene expression *in vitro* including the response to bacterial lipopolysaccharide (LPS). This was to mimic the situation in the postpartum bovine uterus in which most cows develop an initial bacterial infection following calving [28,29]. Six candidate genes were selected for various reasons. Three (*BRINP1*, *IL17RB* and *CRISPLD2*) were taken from the top 10 list of genes whose endometrial expression correlated most highly with circulating urea. *BRINP1* has antiproliferative effects on cultured cells and can modulate the activities of the key receptors ERA, RARA and AR [32,33]. *IL17RB* encodes a cytokine receptor which binds IL-25 (IL17E) to mediate Th2 immune responses [34,35]. *CRISPLD2* encodes a lipopolysaccharide-binding serum protein thought to have an anti-inflammatory function [36,37]. *IGF1R* was selected because we previously found a negative correlation between endometrial *IGF1R* expression measured by qPCR and the circulating urea concentration [27]. *HSP5A* (also known as *GRP78*) encodes a heat shock protein found within the endoplasmic reticulum which is important for protein folding and assembly and is also an indicator of cellular stress which is rapidly up-regulated in response to chemical injury [38,39,40]. Finally S100A8 belongs to the S100 family of calcium-binding proteins which have a variety of actions in innate immunity including anti-microbial activity [41]. We have previously demonstrated strong expression of *S100A8* in endometrial epithelial and stromal cells and have also shown that LPS caused a rapid up-regulation of *S100A8* mRNA and protein in cultured bovine endometrium [42,43].

## 2. Experimental Section

### 2.1. Animals and Management

For Study 1, the *in vivo* experiment, procedures were carried out under license in accordance with the European Community Directive, 86-609-EC. Full details of the treatments have been reported previously [31]. In brief, multiparous Holstein-Friesian cows with a mean parity of 4.7, an average previous lactation yield of 6477 ± 354 kg and a normal calving were used. All animals received the same pre-calving diet comprising *ad libitum* access to grass silage with 2 kg/day citrus pulp introduced 2 weeks before the expected calving date. Cows were blocked 2 weeks prior to expected calving according to parity, body condition score and previous yield and were randomly allocated to 2 treatments (each *n* = 6 cows) designed to produce mild or severe NEB (MNEB or SNEB). From day 2 after calving, MNEB cows were fed grass silage containing 13.6% crude protein *ad libitum* with 8 kg/day of a 20.2% crude protein dairy concentrate and milked once daily. SNEB cows were fed a limited diet of 25 kg/day silage with 4 kg/day concentrate and milked three times daily. The chemical composition of silage and concentrate offered was the same across treatment groups and full details of the diets have been published previously [44]. Daily measurements of milk yield, milk composition, dry matter intake (DMI), body weight, and dietary energy intake were used to calculate EB, based on the French net energy for lactation (NE_L)_ system. Net EB was calculated as UFL/day in which 1 unité fourragère lait (UFL) is the NE_L_ equivalent of 1 kg of standard air-dry barley as described previously [45].

Samples of endometrium were collected from all cows following slaughter at 14 ± 0.4 days postpartum as described below. Array data from one MNEB cow failed the inter-array quality control analysis (see below) so this animal was excluded from all analyses, leaving 5 cows in the MNEB group.

### 2.2. Blood Sampling and Metabolite Assays

Blood samples were collected after morning milking (08:00 h) by jugular venepuncture twice weekly throughout the 2 week treatment period up to and including the day of slaughter. Samples were collected into lithium-heparin primed vials and were immediately placed on ice before centrifugation at 2000× g for 10 min. Plasma was decanted and stored at −20 °C for subsequent analysis for urea, glucose, NEFAs and BHB using the appropriate kit and an ABX Mira auto-analyzer (ABX Mira, Cedex 4, France). IGF1 was analysed using an OCTEIA IGF-I Kit (IDS, Tyne and Wear UK) and insulin using a solid phase radioimmunoassay (Coat-a-Count, Diagnostic Products, Los Angeles, CA, USA) as described previously [46]. Concentrations of the PGF_2α_ metabolite PGFM were quantified using a charcoal-dextran RIA method as described previously [47]. Blood haematology parameters were also determined in unclotted (EDTA treated) whole blood samples using an electronic particle Nihon Kohden haematology analyser (Celltac MEK-610K, Nikon-Kohdon, Tokyo, Japan).

### 2.3. Uterine Tissue Collection and RNA Isolation

The uterus was opened and samples of intercaruncular endometrial tissue weighing approximately 1 g were dissected from the mid portion of the previously gravid horn approximately 1 cm anterior to the bifurcation of the uterus. These were rinsed in RNase free phosphate buffer, snap-frozen in liquid nitrogen and stored at −80 °C. Total RNA was prepared from 200 to 300 mg of fragmented frozen endometrial tissue and homogenized in TRI reagent (Molecular Research Centre Inc, Cincinnati, OH, USA). RNA concentration and purity were determined using the NanoDrop ND-1000 spectrophotometer (NanoDrop Technologies Inc., Wilmington, DE, USA). RNA integrity was confirmed for all samples using automated capillary gel electrophoresis on a Bioanalyzer 2100 with RNA 6000 Nano Labchips according to manufacturers’ instructions (Agilent, Waldbronn, Germany). Additional samples of uterine tissue (both caruncular and inter-caruncular) from the body, mid-region and tip from both horns were fixed in 4% paraformaldehyde and embedded in paraffin for subsequent histological analysis as described below.

### 2.4. Microarray, Correlation and Pathway Analysis

Microarray hybridization and data acquisition were carried out in ARK-Genomics (Roslin Institute, Edinburgh, UK) using 24 K Affymetrix GeneChip Bovine Genome Arrays based on the established ARK-Genomics protocols (http://www.ark-genomics.org/protocols). The acquired data were analyzed using S+ ArrayAnalyzer 2.1 built in S-Plus Enterprise Developer 7.0 software package (Insightful Corp, Seattle, Washington, USA). The probe level expression data generated by the scanner (CEL files) were imported into the ArrayAnalyzer. They were filtered out if the detection was absent or if the pairs used were less than 7 (11 pairs in total). The probe pairs were summarized into a single value per gene using Robust Multichip Analysis (RMA) with a primary Quantiles normalization. After this filtration and summarization, about 20,000 probes/genes were available. The inter-array quality control analysis using MvA and box plots showed that the sample from one cow did not meet the requirements so this animal’s data were excluded from all the analyses. The summarized data were further normalized with Median Inter-quartile Range (IQR). The correlation between the mean plasma urea concentration for each cow and normalized uterine gene expression values was established using a Pearson correlation via the function built in MS Excel 2013 and statistical significance of the correlation was examined using a two tailed t-test. The significantly correlated genes at *p* < 0.05 were loaded into the Affymetrix website for annotation (http://www.affymetrix.com). The GEO-deposited data can be accessed at: http://www.ncbi.nlm.nih.gov/geo/query/acc.cgi?acc=GSE15544. The annotated genes were organized using Entrez Gene combined with gene symbols as identifiers and correlation coefficients and their P-values as observations. They were loaded into Ingenuity Pathway Analysis (IPA) V7.5 software server (Ingenuity Corp, Redwood City, CA, USA) for mapping into relevant functional groups and pathway analysis.

### 2.5. Histological Analysis

Paraffin embedded samples were sectioned at 10 µm and mounted on glass slides. Endometrial sections of each animal were stained with haematoxylin and eosin (H&E) as described previously [43]. The H&E stained specimens were used to assess the degree of uterine inflammation as described by Bonnett *et al*. [48]. Inflammatory cells were subdivided into segmented (neutrophils) or mononuclear cells (macrophages and lymphocytes). The number of segmented and mononuclear cells were the mean of 3 sections per horn (gravid and non-gravid), taken in each case from the uterine tip and a caruncular region and an intercaruncular region from the mid region under a Nikon 187,907 light microscope (×400 magnification). Those in epithelium were expressed as number per graticule length and those in stroma as the average number of cells per 10 µm^2^. The number of lymphocytic foci was counted in stroma under a light microscope (×100 magnification) and was expressed as the number per section.

### 2.6. Primary Bovine Endometrial Cell Culture

For Study 2, apparently healthy bovine uteri in the early luteal phase of the oestrous cycle as determined by the physical appearance of the corpus luteum were obtained from the local abattoir immediately after slaughter and returned to the laboratory on ice for processing within 2 h. On each occasion endometrial epithelial and stromal cells were isolated from the endometrium and then cultured using methods as described previously [42]. Briefly, strips of endometrial tissue were chopped with a tissue chopper and then digested for 90 min at 37 °C in a medium containing 100 mg of bovine serum albumin (BSA; Sigma), 50 mg of trypsin III (Worthington) and 50 mg of collagenase A (Roche) per 100 mL of Hanks’ balanced salt solution (HBSS; Sigma). Digested tissue was filtered through 100 µm sterile cell strainers (BD Falcon) and then washed twice, first by re-suspending in HBSS containing 10% fetal bovine serum (FBS; Sigma) and 3 µg/mL of trypsin inhibitor (Sigma) followed by centrifugation at 100× g and 10 °C for 10 min. Cell sediments were pooled together for each cow sample and the cell count and cell viability evaluated by staining with trypan blue (Sigma). The isolated mixed endometrial epithelial and stromal cells were re-suspended in growth medium (GM) which comprised Dulbecco’s Modified Eagle Medium: Nutrient Mixture F-12 (DMEM/F12; Sigma) containing 10% FBS and 1% antibiotic solution (100 IU/mL penicillin + 100 µg/mL streptomycin; Sigma). Cells were allocated at 5 × 10^5^ cells/well to 24-well plates (Nunc) and cultured at 37 °C and 5% CO_2_ for 8 days to reach confluence.

### 2.7. Endometrial Cell Culture Validation

Cell cultures were validated using immunocytochemical staining to identify specific cell types as described previously [42]. Endometrial epithelial cells stained positive for cytokeratin; stromal cells were positive for vimentin whereas immune cells (e.g., macrophages and granulocytes) stained positive for CD172. The respective primary monoclonal mouse antibodies used were: anti-human cytokeratin- clone AE1/AE3 (Dako), anti-vimentin-clone V9 (Dako) or anti-CD172a (DH59B; Monoclonal Antibody Center VM&P, Washington State University, Pullman, WA, USA). The relative proportions of each cell type after 8 days of culture were evaluated using image analysis software (ImageJ version 1.44; Research Services Branch, NIMH/NIH, Bethesda, MD, USA). Only cultures with an epithelial to stromal cell ratio of approximately 9:1 at day 8 of culture when the urea experiments were performed in addition to a negligible presence of contaminant immune cells (<0.001%) were used for further studies.

### 2.8. Cell Culture Treatment with Urea and LPS

The *in vitro* experiment (Study 2) was performed in a 4 × 2 factorial design in 24-well culture plates. There were four urea treatment groups each of six wells, half of which also received an LPS treatment. The entire procedure was repeated on four separate occasions each utilizing endometrial cell cultures established from a different cow, with each cow representing one batch. Once the cultures had reached confluence (day 8), they were treated with four concentrations (0, 2.5, 5.0 and 7.5 mmol/L) of urea in serum-free medium (SFM) which comprised of 1.125 g BSA and 1 mL Insulin-Transferrin-Sodium Selenite (ITS; Sigma) per L of DMEM/F12. After an initial 2 h equilibration period with the urea present, half of the wells in each urea treatment group were also treated with 100 ng/mL LPS (*E.coli* serotype 026:B6; Sigma) for a further 24 h while the remainder were not. This provided three wells for each treatment and challenge combination and these were pooled for RNA extraction after removal of the culture medium. Total RNA was isolated from the treated cultures using the RNeasy Mini spin column method (Qiagen) following the supplied protocol. The concentration and purity of RNA samples were determined using a ND-1000 NanoDrop spectrophotometer (NanoDrop Technologies) while RNA integrity was confirmed by agarose gel electrophoresis.

### 2.9. Endometrial Cell Viability Assay

Bovine endometrial cell viability following treatment with urea and LPS was determined using the CellTiter 96 AQueous One Solution Cell Proliferation Assay (Promega) in accordance with the supplied protocol based on the 3-(4, 5-dimethylthiazol-2-yl)-5-(3-carboxymethoxyphenyl)-2-(4-sulfophenyl)-2H-tetrazolium (MTS) compound. About 50,000 mixed bovine endometrial epithelial and stromal cells were allocated per well in 32 wells of a 96-well plate and cultured for 8 days. Growth medium was removed from the cultured cells and the urea treatments (0, 2.5, 5.0 and 7.5 mmol/L) allocated to 8 wells per treatment group followed by incubation for 2 h. Each urea treatment group was then divided into two: four wells were treated with 100 ng/mL LPS (*E. coli* serotype 026:B6; Sigma) for 24 h while four were not. A cell viability assay was performed and the measured absorbance at 490 nm was evaluated as a direct proportion of the viable cells in culture.

### 2.10. Quantitative Reverse Transcription PCR (qRT-PCR) Analysis and Data Normalization

Six candidate genes with known functions whose expression in the endometrium correlated with plasma urea concentration in dairy cows (*BRINP1*, *CRISPLD2*, *HSPA5*, *IGF1R*, *IL17RB*, *S100A8*) in addition to *RN18S1*, used as an endogenous reference gene, were selected. Gene expression in the treated endometrial cell cultures was measured by qRT-PCR using methods validated in our laboratory [42]. Oligonucleotide primers were designed for the genes (Table 1) using Primer3 version 4.0 [49] and reference sequence templates derived from the GenBank database (NCBI). Primer specificity to the target gene was evaluated using Primer-BLAST (NCBI). The quality of primers was assessed using OligoAnalyzer version 3.1 (Integrated DNA Technologies) before they were synthesized by the manufacturer (Eurofins MWG Operon). Primer specificity was validated by PCR-gel electrophoresis.

**Table 1:** Details of the genes assessed by quantitative RT-PCR.

Gene Symbol	Primer Sequence 5′→3′	Product Size (bp) *	GenBank Accession No.
*RN18S1*	F: CGGCGACGACCCATTCGAAC	99	NR_036642.1
	R: AATCGAACCCTGATTCCCCGTC		
*S100A8*	F: TGCCATTAACTCCCTGATTGAC	179	NM_001113725.1
	R: TAATTCCACCATCCTGATTGAT		
*HSPA5*	F: GGTATTGAAACTGTGGGAGGTG	119	NM_001075148.1
	R: AAGGTGATTGTCTTTCGTCAGG		
*IGF1R*	F: GATCCCGTGTTCTTCTACGTTC	101	NM_001244612.1
	R: AAGCCTCCCACTATCAACAGAA		
*IL17RB*	F: AAAGCCACTTCCAGTCCTACAG	179	NM_001083467.1
	R: ACCGTCCTCATTCATATTTGC		
*BRINP1*	F: ACTGGAGCAATCAAGGTCACA	173	NM_001015669.1
	R: GCCGACTGGACGAACTTCT		
*CRISPLD2*	F: ACTGAAACGGACGACATGAAC	175	NM_001100299.1
	R: TGGACCCTTTACACTTGTCCTT		

F (forward); R (reverse); * base pairs.

For each gene, qRT-PCR assay was initially optimized and then performed using the CFX96 Real-Time Thermal Cycler (Bio-Rad) and the KAPA SYBR FAST qPCR Kit (Kapa Biosystems). Each 1 µg total RNA sample was first treated with an RNase-free DNase (Promega) and then reverse-transcribed to complementary DNA (cDNA) using the GoScript Reverse Transcription (RT) System (Promega). Assay standards were prepared from PCR gene products which were purified using the the QIAquick PCR purification kit (Qiagen). All assays for each gene were run in duplicate in the same reaction using 50 ng of cDNA sample together with the no template control (NTC) and ten known concentrations of the gene standard ranging from 1 × 10^1^ to 1 × 10^−8^ ng/mL. The absolute mRNA expression values for each gene were calculated by comparing the threshold cycle (Cq) values of the unknown samples to that of the known standard curve using the Bio-Rad CFX Manager software version 3.1.

The reference gene *RN18S1* was evaluated for stability under the experimental conditions. Analysis (linear mixed-effects model) showed that the expression of *RN18S1* in bovine endometrial cells was not altered by either urea or LPS treatments alone or in combination (data not shown). The mRNA expression values of the measured genes were therefore normalized by dividing the sample value for each gene with its corresponding value for *RN18S1*. The values were presented as relative gene expression (in arbitrary units) with respect to the *RN18S1* measured in the same samples.

### 2.11. Additional Data Analysis

In Study 1, Pearson correlation analysis was performed between the final EB value, the various blood hormone and metabolite concentrations and the white blood cell count measured at the time closest to slaughter and the measurements made of the different immune cell populations in the endometrium of each cow. A Benjamini Hochberg false discovery rate correction was employed to correct the P values. In Study 2, four batches of cell culture were established with each batch using endometrial cells derived from the uterus of one cow. The cells in each batch were treated with either control (0) or one of three concentrations of urea (2.5, 5.0 or 7.5 mmol/L) in the absence or presence of an LPS challenge. On each occasion there were three wells for each treatment and challenge combination and these were pooled for RNA extraction. Data were analysed using ANOVA with randomised block design via a linear mixed effect model built in IBM SPSS Statistics for Windows, Version 20.0 (Armonk, NY: IBM Corp.). The effects of urea and LPS on gene expression were taken as fixed effects and cow (batch) as a random effect. Results were considered significant when *p* < 0.05.

## 3. Results

### 3.1. Study 1: in Vivo Microarray Experiment

The overall mean plasma urea concentrations in the first 2 weeks postpartum were 5.4 ± 0.35 mmol/L in the SNEB cows (*n* = 6) and 4.35 ± 0.55 mmol/L in the MNEB group (*n* = 5), with an overall range from 3.2 to 6.6 mmol/L between animals. These values overlapped and did not differ between treatment groups. As reported previously, the cows on the SNEB treatment had a worse EB status associated with increased circulating concentrations of NEFA and BHB but reduced concentrations of IGF1 and glucose [31,46]. The EB calculations for individual cows were correlated with circulating concentrations of glucose, NEFA, BHB and urea. Whereas the concentrations of NEFA, BHB and glucose were strongly related both to each other and to circulating IGF1, those of urea were not. Urea was, however, the only metabolite to show a positive correlation with the circulating PGFM value (Table 2).

With respect to the immune cell status of the cows in both blood and endometrium, the plasma concentrations of IGF1, insulin, glucose, NEFA and BHB were all significantly correlated with the white blood cell count. In accord with this, glucose and BHB were negatively and positively correlated respectively with the numbers of segmented cells present in the luminal epithelium, and IGF1, insulin, glucose, NEFA and BHB all showed significant relationships with the numbers of lymphocytic foci and/or the monocyte population of the endometrial stroma (Table 2). In contrast, plasma urea was not related to any measurement made of the immune cell population in either blood or endometrium.

There were, however, 1310 probes from the microarray data whose expression in endometrium correlated with the mean plasma urea concentration of each cow at *p* < 0.05. From this total 781 genes were mapped (listed in Table S1) and their biological functions were analysed using Ingenuity Pathway Analysis (IPA) (Table 3). The main disease processes identified were endocrine system disorder and haematological disease whereas the top two biological functions were cellular function and maintenance and cellular growth and proliferation (all *p* < 0.001). The top four canonical pathways were corticotrophin releasing hormone signaling, coagulation system, retinoic acid mediated apoptosis signaling and LXR/RXR activation. Although haematological disease was identified as significant, there were no significant correlations of the plasma urea concentration with the populations of immune cells (neutrophils, macrophages and lymphocytic foci) measured in the epithelium or stroma of the individual cows (Table 2).

**Table 2:** Matrix of Pearson correlations between energy balance and circulating metabolite and hormone concentrations at 14 ± 0.4 days postpartum with the circulating white blood cell count and the uterine inflammatory cell population (*n* = 11 cows) #.

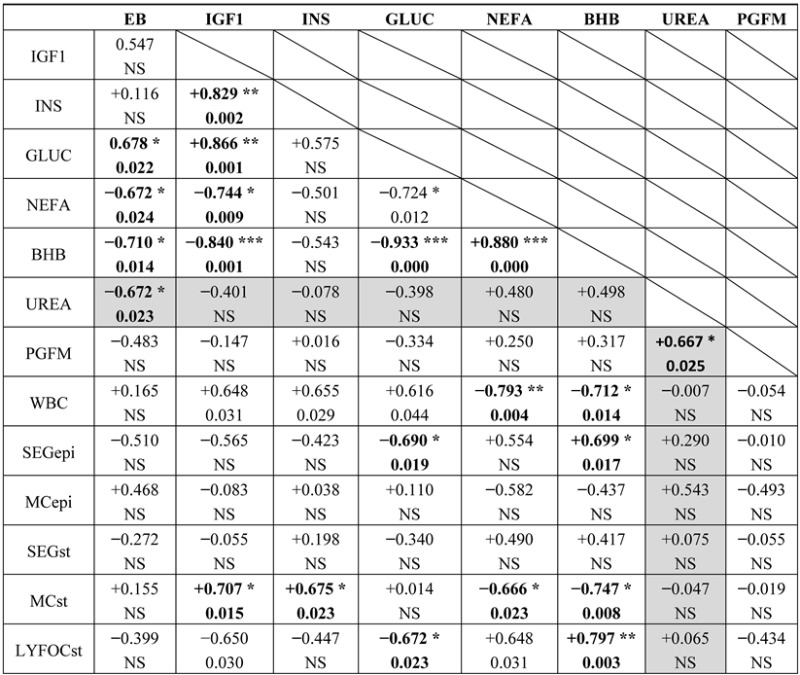

# Results have been combined for the 5 cows on the MNEB and 6 on the SNEB treatment groups. The blood values used were those taken pre-slaughter. EB, energy balance; INS, insulin; GLUC, glucose; WBC, circulating white blood cells; SEGepi and SEGst, segmented cells in uterine epithelium and stroma; MCepi, and MCst mononuclear cells (macrophages and lymphocytes) in uterine epithelium and stroma; LYFOCst, No. lymphocytic foci in stroma. The Pearson correlation is shown above. The actual *p* value is given below. Using a Benjamini Hochberg false discovery rate correction for 13 × 13 comparisons, adjusted 5% significance is at *p* = 0.025 and adjusted 1% significance is at *p* = 0.005, shown in bold. NS, not significant.

**Table 3:** Ingenuity Pathway Network Analysis of the genes significantly correlated with mean plasma urea concentrations showing the top 4: (1) disease processes and biological functions; (2) canonical pathways and (3) network functions.

*P* Value	No. Genes	Disease Processes and Biological Functions
<0.001	32	Endocrine system disorders
<0.001	28	Haematological disease
<0.001	49	Cellular function and maintenance
<0.001		Cellular growth and proliferation
	**Ratio ^a^**	**Canonical Pathways**
0.01	10/136	Corticotrophin releasing hormone signalling
0.016	5/38	Coagulation system
0.022	5/68	Retinoic acid mediated apoptosis signalling
0.026	10/131	LXR/RXR Activation
**Score**	**Focus Molecules**	**Network ^b^**
43	29	Endocrine system development, lipid metabolism, molecular transport
40	27	Cell-to-cell signaling and interaction, tissue development
38	26	Lipid metabolism, molecular transport, small molecule biochemistry
38	26	Cell-to-cell signaling and interaction, haematological system development and function, haematopoiesis

^a^ The number of genes in the list of DEGs that participate in the canonical pathway divided by the total number of genes that are known to be associated with the pathway in the Ingenuity knowledge base; ^b^ A higher network score corresponds to a lower probability of finding the observed number of the DEGs in a given network by chance.

**Figure 1:**
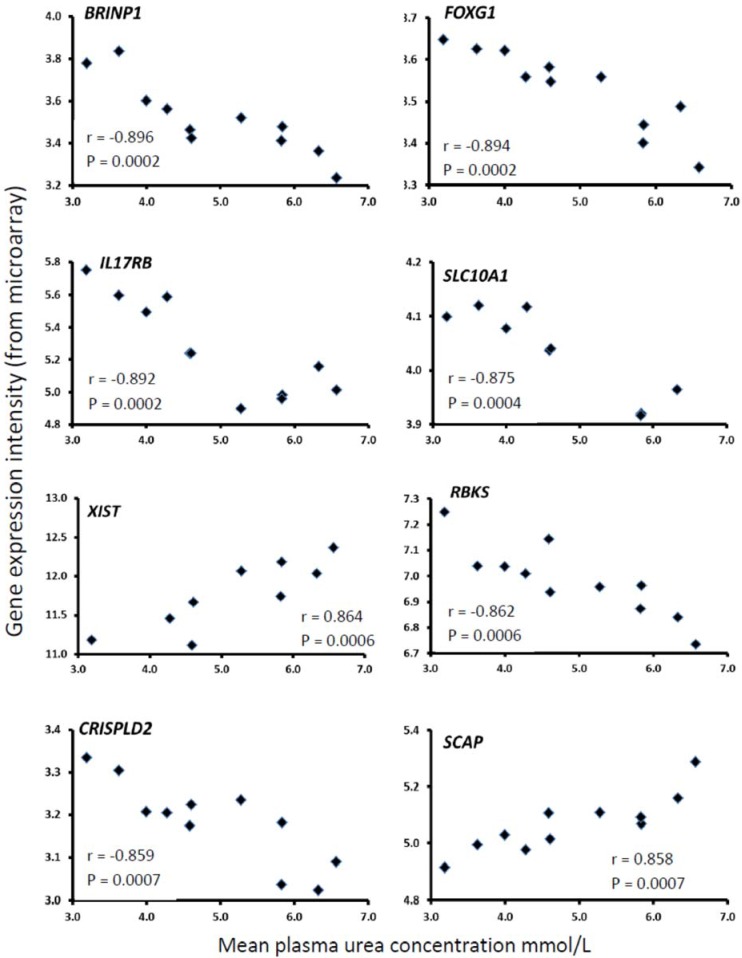
Scatter plots showing the relationships between the mean plasma urea concentration from each of 13 cows and the gene expression level measured in their endometrium at 2 weeks postpartum using microarray analysis, normalized by overall expression within the chip.

The top 10 genes whose expression in the endometrium was most highly correlated with plasma urea concentrations are summarized in Table 4 and for eight of these genes the relationship is illustrated in Figure 1. In most cases the relationship was negative, with gene expression decreasing as plasma urea increased but *SCAP, XIST* and *ACBD5* showed a positive relationship. The putative functions of these genes varied considerably including potentially important regulatory roles in cellular physiology and immune function. The protein encoded by *BRINP1* is thought to be a critical regulator of tumorigenesis: in cultured breast cancer cells it can modulate the activities of the key receptors ERA, RARA and AR [33] whereas in a bladder tumour cell line it clustered with several proteins in the urokinase-plasminogen pathway involved in inflammation [50]. *FOXG1* encodes a protein from a distinct subfamily of the forkhead box O (FOXO) family of transcription factors. These regulate the expression of genes in cellular physiological events including apoptosis, cell-cycle control, glucose metabolism and oxidative stress resistance. FOXG1 itself has been associated with the PI3K and TGFB/Smad signalling pathways [51]. *IL17RB* encodes a cytokine receptor which binds IL-25 (IL17E) to mediate Th2 immune responses [35]. *ACBD5* functions in the transport and distribution of long chain acyl-Coenzyme A in cells. *SLC10A1* encodes a protein belonging to the sodium/bile acid co-transporter family [52]: as bile acids are the catabolic product of cholesterol metabolism, it is important for cholesterol homeostasis. *SRRM2* plays a role in RNA splicing and *RBK5* is involved in ribose metabolism. *XIST* functions in early developmental processes in mammalian females to transcriptionally silence one of the pair of X chromosomes. *CRISPLD2* encodes an LPS-binding serum protein thought to have an anti-inflammatory function [37]. *SCAP* plays a crucial role in regulating the LDL receptor and is involved in inducing macrophages to form foam cells [53].

**Table 4:** Summary information on the top 10 genes expressed in bovine endometrium which were most highly correlated with the plasma urea concentration plus an additional 3 genes selected for the follow-up study.

Gene Symbol	Unigene ID	Gene Title	Entrez Gene	r	P
**Top 10 Genes**					
*BRINP1* #	Bt.35142	Bone morphogenetic protein/retinoic acid inducible neural-specific 1	538990	−0.896	0.0002
*FOXG1*	Bt.66213	Forkhead box G1	516947	−0.894	0.0002
*IL17RB*	Bt.24532	Interleukin 17 receptor B	533605	−0.892	0.0002
*ACBD5*	Bt. 87957	Acyl-coenzyme A binding domain containing 5	353160	+0.883	0.0003
*SLC10A1*	Bt.51814	Solute carrier family 10 (sodium/bile acid co-transporter family), member 1	532890	−0.875	0.0004
*SRRM2*	Bt.22353	Serine/arginine repetitive matrix 2	539515	−0.869	0.0005
*XIST*	---	X (inactive)-specific transcript	338325	+0.864	0.0006
*RBKS*	---	Ribokinase	513276	−0.862	0.0006
*CRISPLD2*	Bt.55503	Cysteine-rich secretory protein LCCL domain containing 2	505329	−0.859	0.0007
*SCAP*	Bt.18085	SREBF chaperone	507878	+0.858	0.0007
**Additional Follow-up Genes Used in Study 2**					
*S100A8*	Bt. 9360	S100 calcium binding protein A8	616818	+0.624	0.04
*HSPA5+*	Bt.65104	Heat shock 70 kDa protein 5 (glucose-regulated protein, 78 kDa)	415113	+0.580	0.06
*IGF1R*	Bt.12759	Insulin-like growth factor 1 receptor	281848	+0.609	0.05

# Previously named *DBC1*, Deleted in bladder cancer 1; + Also known as *GRP78*.

The four top networks identified were involved in cell signalling, lipid metabolism and haematopoiesis. The components of each network are given in Table 5. Network 1 was “Endocrine system development, lipid metabolism and molecular transport”. Some genes within this network encoded proteins involved in the regulation of mitosis (*ARPP19*, *SMC3*) whereas XIST, as mentioned above, is important for inactivation of one X chromosome in females. The network included several genes encoding proteins which are components of the mediator complex which is involved in the regulation of gene transcription (*MED17*, *MED22*, *MED25*, *MED26*). A number of other genes play a role in altering mRNA stability (*HADHB*, *MATR3*) and in RNA degradation (*RNASE1*). *HADHB* is also involved in the beta-oxidation of long chain fatty acids. Network 1 also contained some genes involved in the innate immune system: *CEPBP* encodes a transcription factor involved in the regulation of genes involved in immune and inflammatory responses; *CXCL5* encodes a chemokine which recruits neutrophils to promote angiogenesis and remodel connective tissues whereas *DEFB1* encodes an antimicrobial peptide implicated in the resistance of epithelial surfaces to microbial colonization. Two other interesting genes identified were *NR3C1* and *POMC*. *NR3C1* encodes the glucocorticoid receptor, which can function both as a transcription factor that binds to glucocorticoid response elements to activate gene transcription and as a regulator of other transcription factors. It is involved in inflammatory responses, cellular proliferation, and differentiation in target tissues. *POMC* encodes a polypeptide hormone precursor that is synthesised mainly in corticotroph cells of the anterior pituitary but also in peripheral tissues [54]. The precursor protein undergoes extensive tissue-specific, post-translational processing yielding up to ten biologically active peptides which are involved in diverse cellular functions with roles in steroidogenesis, pain, energy homeostasis, melanocyte stimulation and immune modulation. Amongst these αMSH has immunmodulatory and antimicrobial activity [55].

**Table 5:** Molecules in each of the top 4 networks and canonical pathways in endometrium of 11 postpartum cows identified by IPA analysis as being correlated with their mean plasma urea value.

Network	Molecules in Network
Endocrine system development, lipid metabolism, molecular transport	Alpha tubulin, *ARPP19*, *ASCC2*, *CEBPB* (includes EG:1051), *CRTC1*, *CXCL5*, *DEFB1*, *FASTKD1*, FASTKD5, *HADHB*, Ige, Interferon alpha, Lh, *MATR3*, *MED17*, *MED22* (includes EG:20933), *MED25* (includes EG:292889), *MED26* (includes EG:306328), Mediator, *MEIS1*, *MFSD2A*, *MLL2*, *NR3C1*, *PALB2*, *PCTP*, *POMC*, *RAD51AP1*, RNA polymerase II, *RNASE1*, *RPL12*, *SMC3*, *TMEM176A*, *TNS4*, *UBE2O*, *XIST*
Cell-to-cell signaling and interaction, tissue development	Aconitase, *ADAM9*, *APEH*, *CBR1*, *CDH16*, *CHMP3*, *CNKSR3*, *DOCK1*, *ELMO3*, *EPHA7*, *ERK1/2*, *FEZF1*, *FXN*, *HADH*, *HERC2*, *JAM*, Laminin, Lfa-1, *LGI1*, *LLGL2*, Mac1, *MLLT4*, *MVP*, *NAA15*, *PCBP2*, *RABAC1*, Rap1, Rap, *RAP1A*, *RAPGEF2*, *RASIP1*, *SNX15*, *TMEM176B*, *UBA5*, *VTA1* (includes EG:292640)
Lipid metabolism, molecular transport, small molecule biochemistry	*ABCG4*, *AKR1C4*, Angiotensin II receptor type 1, *APOA5*, C/ebp, *CHST14*, *CLEC4G*, *CPN1*, *GALNT2*, *GC*, *HDL*, HDL-cholesterol, *HIVEP2, HNF1B*, *HSD11B1*, *IL17RB*, *IP6K2*, *IRAK1BP1*, *KPNA4*, *MAP4K2*, N-cor, NCOR-LXR-Oxysterol-RXR-9 cis RA, NFkB (complex), *NR1H2*, *OTUB2*, *PEPCK*, *PPP1R13L*, *RTKN*, Rxr, *SERPINF1*, *SERPINF2*, *SLC10A1*, *STMN2*, *TFG*, *ZFAND6*
Cell-to-cell signaling and interaction, haematological system development and function, haematopoiesis	*ABI1*, Akt, *ASXL2*, *BATF, BBS5*, *BCOR*, *C1q*, Complement component 1, Creatine Kinase, *CREM*, *CYP19*, Cytoplasmic Dynein, *DYNLRB2*, Fibrinogen, *FKHR*, *FOXG1*, *GDF9*, *GRB10*, *HOXD10*, *MLLT1*, *MLLT10*, *MPDZ*, *NDEL1*, *NFIL3*, *PCGF1*, *PCM1*, *PHF1*, *PTX3*, *RAB40B*, *SERPING1*, *SPINK1*, *TDGF1*, *TFPI*, Trypsin, *TTC3*
**Canonical Pathways**	**Molecules in Canonical Pathway**
Corticotropin Releasing Hormone Signalling	*ATF4*, *GNAS*, *GUCY2C*, *MAPK12*, *MEF2C*, *POMC*, *PRKCD*, *RAP1A*, *RAF1*, *VEGFA*
Coagulation System	*A2M*, *F3*, *PLAUR*, *SERPINF2*, *TFPI*
Retinoic acid Mediated Apoptosis Signaling	*CFLAR*, *IFNAR1*, *FADD*, *PARP10*, *TIPARP*
LXR/RXR Activation	*ABCG4*, *APOA5*, *C3*, *GC*, *HADH*, *IRF3*, *NR1H2*, *S100A8*, *SERPINF1*, *SERPINF2*

Network 2 was entitled “Cell-to-cell signalling and interaction, tissue development”. A number of genes (*CDH16*, *ADAM9*, *CHMP3*, *DOCK1*, *ELMO3*, *EPHA7*, *FEZF1*, *LLGL2*, *RAP1A*) encode proteins which have roles in tissue development, cell-cell and cell-matrix interactions, cell migration and membrane turnover. For example, *ADAM9* is involved in shedding membrane-anchored heparin binding EGF-like growth factor, *CHMP3* sorts transmembrane proteins into lysosomes, *DOCK1* and *ELMO3* are both involved in phagocytosis and cell migration. Both *SNX15* and *VTA15* encode proteins involved in intracellular trafficking. *CBR1* encodes a NADPH-dependent oxidoreductase which has wide specificity for carbonyl compounds including prostaglandins. *FXN* encodes a mitochondrial protein regulating iron transport whereas *HADH* functions in the mitochondrial matrix in the beta-oxidation pathway. *PCBP2* encodes a multifunctional protein involved in RNA binding.

Network 3 was called “Lipid metabolism, molecular transport, small molecule biochemistry”. Proteins encoded by several genes within this network are involved in various aspects of lipid metabolism. *ABCG4* is thought to play a role in cholesterol transport, *APOA5* is an apolipoprotein which helps to regulate plasma triglyceride levels, as does *GALNT2*. *NR1H2* encodes the liver X receptor, LXRB, which is important for macrophage function and lipid homeostasis, regulating the metabolism of various lipids including cholesterol and bile acids. *SLC10A1* is involved in the breakdown of bile acids and thus is also important for cholesterol homeostasis. *AKR1C4* is a member of the aldo/keto reductase superfamily with roles in the inter-conversion of steroids and prostaglandins between active and inactive forms. Similarly *HSD11B1* encodes an enzyme which catalyzes the conversion of cortisol to the inactive form cortisone. *GC* encodes a member of the albumin family which binds and transports vitamin D and its metabolites. Some other genes in this network are involved in immune function including *CLEC4G* (T cell responses), *IL17RB* (cytokine receptor) and *IRAK1BP1*, a gene associated with the interleukin 1 receptor. *SERPINF1* and *SERPINF2* encode serpin peptidase inhibitor, clade F (alpha-2 antiplasmin, pigment epithelium derived factor), members 1 and 2 respectively. SERPINF1 strongly inhibits angiogenesis whereas SERPINF2 is a major inhibitor of plasmin, which degrades fibrin and various other proteins thus having an important role in regulating the blood clotting pathway.

Network 4 was entitled “Cell-to-cell signalling and interaction, haematological system development, haematopoiesis”. It includes a number of transcriptional regulators including *ASXL2*, *BATF*, *BCOR*, *CREM*, *FKHR* (also known as *FOXO1A*), *FOXG1*, *HOXD10*, *MLLT10*, *NFIL3*, *PCGF1* and *PHF1*. Within this list *ASXL2*, *PCGF1* and *PHF1* encode proteins belonging to the polycomb group which are involved in the epigenetic regulation of gene expression during development and differentiation. C1q is part of the complement system while *SERPING1* encodes a protein involved in regulation of the complement cascade. *PTX3* is also involved in innate immunity. *CYP19* encodes a cytochrome P450 which catalyses oestradiol synthesis. *NDEL1* and *PCM1* are both involved in cytoskeleton reorganization. *GRB10* encodes a growth factor receptor binding protein which interacts with both insulin and IGF receptors to influence their downstream signalling. *TDGF1* encodes a TGFB ligand and the TGBF signalling pathway plays numerous roles in cell proliferation, differentiation, apoptosis and migration.

Not surprisingly there was overlap between some of the genes classified into networks and canonical pathways. The most significant canonical pathway was “Corticotropin Releasing Hormone Signalling” which contained both *POMC* and also *VEGFA*, highlighting the activities of melanocortin peptides as important regulatory functions in vascular inflammation [56]. The second pathway related to the “Coagulation System”. *C3* encodes coagulation factor III which enables cells to initiate the blood coagulation cascades. *PLAUR* has a role in plasminogen activation and localized degradation of the extracellular matrix while both *A2M* and *SERPINF2* encode protease inhibitors. SERPINF2 has a major role in regulation of blood clotting. Pathway 3 was entitled “Retinoic Acid Mediated Apoptosis Signalling”: two of the genes identified within it, *CFLAR* and *FADD*, are mainly implicated in regulation of apoptosis while *IFNAR1* encodes an interferon receptor. Canonical pathway 4 “LXR/RXR Activation” contained several genes discussed above which also appeared in Network 3 (*ABCG4*, *APOA5*, *NR1H2*, *SERPINF1*, *SERPINF2).* GC is a vitamin D binding protein while S100A8 is a calcium-binding protein with a role in innate immunity.

### 3.2. Study 2: in Vitro Effects of Urea on Endometrial Gene Expression

Initial tests in the absence of any added cells measured the osmolality and pH of the medium for the four tested concentrations of urea (0, 2.5, 5.0 and 7.5 mmol/L). These were equivalent to 0, 150, 300 and 450 µg/mL. Osmolality increased very slightly from 0.29 ± 0.001 osmol/kg at 0 µg/mL urea to 0.30 ± 0.002 osmol/kg at 450 µg/mL. The pH also increased slightly from 7.5 ± 0.02 to 7.6 ± 0.01 over the same urea range. These values all remained within ranges considered acceptable for cell culture. The effects of the treatments were next tested with respect to cell viability using the CellTiter 96 AQueous One Solution Cell Proliferation Assay (Promega). The absorbance values at 490 nm showed that treatment *in vitro* of mixed bovine endometrial epithelial and stromal cell cultures with four different concentrations of urea with or without exposure to 100 ng/mL LPS for 24 h did not alter the number of viable cells in culture compared to untreated cells (data not shown).

All the candidate genes (*S100A8*, *HSP5A*, *IGF1R*, *IL17RB*, *DBC1* and *CRISPLD2*) and the reference gene *RN18S1* were detectable in the endometrial cultures by qRT-PCR. A comparison of the normalised data showed that treatment with four different concentrations of urea (0, 2.5, 5.0 and 7.5 mmol/L) did not alter the expression of any of the examined genes in cultured bovine endometrial cells (*p* > 0.05, Table 6). Furthermore, there were no significant interactions between the effect of urea and LPS treatment on the mRNA expression of the examined genes (*p* > 0.05, Table 6). As expected, the expression of *S100A8* was up-regulated (*p* < 0.001; *t*-test) in the endometrial cell cultures treated with LPS for 24 h. In contrast, LPS did not alter the expression of *HSP5A*, *IGF1R*, *IL17RB*, *DBC1* and *CRISPLD2* in the treated cultures (Table 6).

**Table 6:** Effects of different concentrations of urea on the mRNA expression of selected genes in bovine endometrial cells with or without 100 ng/mL LPS for 24 h ^#^.

Gene	LPS	Urea (mmol/L)				COMB+
		0	2.5	5.0	7.5	
*S100A8*	-	90 ± 34.8	90 ± 17.9	112 ± 47.8	86 ± 25.4	94 ± 15.2
	+	545 ± 97.3	816 ± 242	795 ± 263	697 ± 225	713 ± 101 **
*HSPA5*	-	449 ± 110	547 ± 152	533 ± 210	517 ± 191	511 ± 76.7
	+	533 ± 171	532 ± 192	523 ± 179	652 ± 285	560 ± 95.7
*IGF1R*	-	40,686 ± 7122	51,365 ± 6018	40,512 ± 8384	47,800 ± 11,001	45,091 ± 3920
	+	56,308 ± 12,006	41,976 ± 9441	40,360 ± 7702	46,944 ± 11,921	46,397 ± 4931
*IL17RB*	-	1019 ± 471	1802 ± 283	2164 ± 923	2111 ± 337	1774 ± 278
	+	3386 ± 898	1613 ± 795	1295 ± 505	2098 ± 486	2099 ± 373
*BRINP1*	-	334 ± 136	405 ± 142	289 ± 114	254 ± 119	320 ± 59.3
	+	340 ± 141	370 ± 148	381 ± 202	224 ± 52.9	329 ± 67.3
*CRISPLD2*	-	3131 ± 1551	3403 ± 1470	3042 ± 1200	2418 ± 710	2998 ± 578
	+	3261 ± 1511	2687 ± 1071	3390 ± 1105	1666 ± 359	2751 ± 519

^#^ Values are calculated as mean relative expression (in arbitrary units) ± SEM with respect to the reference gene *RN18S1* measured in the same samples. Results are combined from four experimental replicates/batches per treatment. There was no significant interaction (*p* > 0.05; ANOVA with randomised block design via a linear mixed-effects model) between the effect of urea and LPS treatment on the expression of any of the genes examined; + Shows the combined effect of LPS treatment across all the urea concentrations combined, *n* = 16. ** Significant difference (*p* < 0.001; *t*-test).

## 4. Discussion

This study has revealed many highly significant associations between plasma urea levels ranging between 3.2 to 6.6 mmol/L and endometrial gene expression during the early postpartum period when the uterus is not only undergoing extensive remodelling but also playing a major role in innate immunity to combat infection. These urea concentrations were all below the range which others have considered as “high” with suggested thresholds of >6.8 [16] or >7.5 mmol/L [10]. Hammon *et al*. [57] confirmed that in cows with blood plasma urea nitrogen level >20 mg/dL (equivalent to 7.1 mmol/L) the uterine fluids also had significantly higher urea levels. Our second experiment did not, however, find any effects of added urea providing concentrations in the range 0 to 7.5 mmol/L on endometrial gene expression *in vitro*, suggesting that an intermediary mechanism(s) must be involved.

The influence of high protein intake and the effect of urea on dairy cow fertility remain ambiguous [7,12,58]. There is also no single measurable metabolite which directly reflects protein status. Rather, multiple parameters are utilised including blood urea nitrogen (BUN), creatinine, total protein, albumin and creatine kinase [11]. A high mean plasma urea concentration was associated with a significant reduction in pregnancy rate in dairy cows [59,60]. In contrast, high plasma urea concentrations due to high levels of dietary nitrogen had no effect on parameters of fertility in other studies [61,62,63]. Moreover, high blood urea and the metabolic indicators of NEB often occur simultaneously in high-yielding cows making it difficult to separate out any individual effects on subsequent fertility [58]. Another problem of interpretation is that both high (>7.5 mmol/L) and low (<4.5 mmol/L) circulating urea concentrations have been associated with reduced fertility. We showed previously that the relationships between plasma urea and fertility altered according to both the age of the cow and the stage of lactation at which the urea levels were measured [10]. This earlier study analysed data from 500 cows from 6 different farms which were fed 17 different diets in which the crude protein content varied from 133 to 228 g/kg dry matter. The differences in plasma urea concentrations between animals were mainly accounted for by diet but body condition score and milk yield were also important. In multiparous cows there was a trend for *lower* pre-calving plasma urea concentrations to be associated with longer intervals to conception whereas in cows calving for the first time, *higher* plasma urea concentrations pre-calving predicted a worse fertility outcome. The plasma urea concentration measured at 7 weeks postpartum was negatively associated with fertility in both age groups. The cows in the present experiment were fed a standard diet based on grass silage and concentrate and the plasma urea concentrations did not exceed 7.5 mmol/L. There was no difference in the plasma urea concentration between cows on the SNEB and MNEB treatments but other blood metabolite and hormone concentrations changed as predicted (elevated BHB and NEFA, reduced glucose and IGF1 for the SNEB cows). This indicates that other currently unknown cow factors affected the circulating urea concentration.

Although the *in vivo* experiment detected many highly significant correlations between circulating urea and endometrial gene expression, the *in vitro* experiment showed that treatment with 0, 2.5, 5.0 and 7.5 mmol/L of urea equivalent to low, medium and high plasma urea concentrations did not alter the mRNA expression of *S100A8*, *HSP5A*, *IGF1R*, *IL17RB*, *DBC1* and *CRISPLD2* in cultured bovine endometrial cells. LPS treatment caused a significant increase (≈8 fold) in the expression of *S100A8* in bovine endometrial cells which confirmed our previous report [42] and showed that the cells were viable and responsive under all the urea concentrations tested. Urea is an osmotically active agent and changes in osmolality have long been known to have major effects on mammalian cells, including cytoskeletal rearrangement, inhibition of DNA replication and apoptosis [64]. For instance, the expression of heat shock proteins (HSPs) altered during cellular response to osmotic stress induced by urea [65,66]. In contrast, this study observed no differences in expression of *HSP5A* and the other examined genes in response to urea. The previously reported effects were, however, generally only apparent with osmolalities above 500–700 mosmol/kg, dependent on cell type [64]. Whereas such levels are reached in normal renal medulla, they are much higher than those which we tested which reflected physiological urea concentrations in plasma of around 300 mosmol/kg. Another study also reported that the expression of several genes involved in cell growth/differentiation (*IGF1*) and apoptosis/cellular stress (*BCL2*, *BAX*) were not altered in bovine endometrial explants at concentrations comparable to this study [22]. The only gene showing a consistent dose-responsive decline in expression as urea increased was *FGF2*, but significance was only reached at a urea concentration of 16 mmol/L which is much higher than normal circulating levels.

Excess dietary protein or urea infusion decreased uterine pH in cows from about 7.1 to about 6.9, and this effect was specific to the uterus [19,20,67]. When polarized bovine endometrial cells in culture were treated with urea this diminished the effect of progesterone in maintaining a pH gradient between apical and basal compartments [8]. In our study, however, the addition of urea did not alter the pH of the culture medium. The culture system we used also differed as cells were not polarized or treated with progesterone. Beltman *et al*. [68] compared parameters between beef heifers in which either a viable or degenerate embryo was found on day 7 post insemination and found no differences in either blood urea or uterine pH. Another possibility is that the effect of an increase in dietary nitrogen may be mediated by ammonia rather than urea [12]. However, the relationship between plasma concentration of urea and ammonia is complex. Elevated plasma concentrations of ammonia and (or) urea due to a high-protein diet, compromised the capacity of oocytes to develop to blastocysts *in vitro* [17]. Ammonia, in similar concentrations measured in the follicular fluid also impaired the *in vitro* growth, metabolism and functions of bovine ovarian granulosa cells [69].

The lack of a direct effect of added urea on endometrial gene expression suggests that the strong correlations between urea and gene expression found in Study 1 was not due to a direct causal relationship. Another possibility is that both were influenced by some other factor. The cows in this study were being managed to induce either mild or severe NEB and this was confirmed by achieving raised concentrations of BHB and NEFA but lower levels of IGF1 between the groups [31]. We also showed previously that the livers in SNEB cows contained significantly more lipid and reduced glycogen [46]. In contrast, plasma urea levels overlapped between the treatment groups; factors other than diet must therefore have been influential. The samples were collected two weeks after calving when the uterus is still undergoing involution and also mounting an innate immune response to bacterial infection [29,70]. Examination here of the genes, networks and pathways associated with circulating urea has shown that these were predominantly associated with tissue turnover, immune function, inflammation and lipid metabolism.

Involution requires a large amount of tissue breakdown and extensive remodelling to restore normal size and architecture. The presence of large numbers of genes encoding transcription factors (e.g., *FKHR*, *FOXG1*, mediator complex genes, *CEPBP*, *NR3C1*), polycomb group genes involved in epigenetic regulation of gene expression (e.g., *ASXL2*, *PCGF1*, *PHF1*), genes involved with RNA turnover and splicing (e.g., *SRRM2*, *RBK5*, *HADHB*, *MATR3*, *RNASE1*), cytoskeleton reorganisation (*NDEL1*, *PCM1*), cell proliferation (*ARPP19*, *SMC3*) and apoptosis (*CFLAR*, *FADD*) whose expression showed a relationship with urea implies a link between circulating urea and the rate of uterine involution. The most highly correlated gene was *BRINP1* which has been shown to influence the rate of proliferation in tumour cell lines, in part by modulating the activities of the steroid and retinoic acid receptors ERA, AR and RARA [33,71]. *BRINP1* expression fell as the urea concentration increased. This is a chicken and egg situation as animals in energy deficit may increase tissue protein catabolism leading to increased urea production [1], thus further work is required to determine whether the rate of tissue breakdown affected the circulating urea level or vice versa. In postpartum cows plasma PGFM concentrations fall steadily from the peak at calving, generally returning to baseline within about 2 weeks [31]. However in some cows with uterine infections PGFM concentrations remain elevated for longer [72]. Bekana *et al*. [73] found a positive correlation between the occurrence of later additional periods of elevated PGFM and the length of time required for uterine involution. This would accord with our finding of a positive correlation between urea and the circulating PGFM concentration for the cows in the present study.

Another relationship highlighted by the array data was between the plasma urea level and aspects of innate immunity and inflammation, with endometrial gene expression generally decreasing as urea levels increased. The postpartum endometrium undergoes an inflammatory response and the innate immune system plays an important role in the elimination of uterine pathogens during this period which influences subsequent fertility [5,70,74,75]. *IL17RB* encodes a cytokine receptor which binds IL-25 (IL17E) to mediate Th2 immune responses [35]. Another important chemokine gene *CXCL5* featured in Network 1. Both are important in the recruitment of neutrophils to promote angiogenesis. *CRISPLD2* encodes a serum protein which can bind LPS, a major structural component of Gram-negative bacterial cell walls, and is thought to have an anti-inflammatory function [36,37]. *DEFB1* and *S100A8* both encode proteins with antimicrobial activity as does αMSH, which is one of the possible proteins produced from POMC. Several genes involved in the complement cascade and blood clotting were present in Networks 3 and 4 and Canonical Pathway 2. These included *C1q*, *C3*, *SERPING1*, *SERPINF1*, *SERPINF2* and *PLAUR*. These findings support an earlier suggestion that feeding cows high dietary protein levels may in part reduce fertility through an impaired uterine inflammatory response [9]. One of the initial responses of the uterus to postpartum infection is a neutrophilic influx into the superficial endometrium with subsequent mobilisation of macrophages, lymphocytes and eosinophils which is associated with vascular congestion and stromal oedema [76]. This was not supported by measurable differences in histological analysis of the endometrium as there were no correlations between the immune cell populations and the blood urea concentration. Histological assessment is, however, likely to be less sensitive at detecting more subtle changes than the gene expression data.

Another potentially interesting association was between plasma urea concentration and aspects of lipid metabolism. We have shown previously that the endometrium from the cows used in the energy balance study contained many lipid droplets which appeared to be associated with the transformation of macrophages into foam cells [75]. We show here that *SCAP* was one of the genes most highly correlated with urea whose expression increased with higher plasma urea levels. Sterol regulatory element binding proteins (SREBPs) are key transcriptional regulators of fatty acid synthesis and cholesterol metabolism and SCAP is an insulin-inducible SREBP cleavage activating protein which plays a key role in cholesterol homeostasis [77]. Macrophages can develop into foam cells during inflammatory stress. If they become overloaded with cholesterol then the SCAP-SREBP2 complex is retained in the endoplasmic reticulum, SREBP2 cannot be processed, the LDL receptor is down-regulated and both cholesterol uptake and *de novo* synthesis are inhibited [53]. Two other top 10 genes associated with plasma urea were *ABCD5*, which is involved in the distribution of long chain acyl-coenzyme A, and *SLC10A1* which encodes a sodium/bile co-transporter. Network 3 identified *ABCG4*, involved in cholesterol transport, *APOA5* and *GALNT2*, both involved in the regulation of plasma triglyceride levels and *NR1H2* which encodes LXRB, the liver X receptor. LXRs are oxysterol-activated nuclear receptors. In macrophages exposed to excess cholesterol LXRs facilitate cholesterol transport out of the cells into HDL particles [78]. Cholesterol is a key component in the synthesis of bile salts by the liver. There is increasing evidence that bile salts act as nutrient signalling molecules which collaborate with insulin in the regulation of hepatic nutrient metabolism [79]. A proteomic study in dairy cows found that a high liver triglyceride content was associated with increased oxidation of saturated fatty acids, oxidative stress and urea synthesis [80]. While these networks and associations require more work to unravel, they do suggest that elevated circulating urea concentrations in cattle may be associated with alterations in cholesterol metabolism and macrophage activity within the postpartum endometrium and it is these rather than the urea concentration which could potentially influence subsequent fertility.

## 5. Conclusions

In summary, identification of signalling factors associated with uterine endometrial function helps to improve our understanding of the underlying mechanisms causing reduced fertility in dairy cows. The expression of many genes in the involuting endometrium which are involved particularly in tissue turnover, immune function, inflammation and lipid metabolism was strongly associated with the plasma urea concentration. The results presented here suggest that the urea effect is not, however, direct on the endometrium. Instead, the variations in circulating urea may be a consequence of metabolic differences particularly associated with lipid metabolism in postpartum dairy cows which may influence fertility through other mechanisms.

## Supplementary Files

Supplementary File 1
