# Supplementary material for: Relationships between Circulating Urea Concentrations and Endometrial Function in Postpartum Dairy Cows
Source: Animals (Basel). 2015 Aug 14;5(3):748–73. doi: 10.3390/ani5030382 (PMC4598704; doi:10.3390/ani5030382)
Supplement: Supplementary File 1 [file animals-05-00382-s001.docx]

**Table S1.** List of genes expressed in endometrium of 11 postpartum dairy cows for which the expression was significantly correlated with the plasma urea concentration.

| **Probe Set ID** | **UniGene ID** | **Gene Title** | **Gene Symbol** | **Entrez Gene** | **r** | **P** |
| --- | --- | --- | --- | --- | --- | --- |
| Bt_23514_1_S1_at | Bt.60794 | alpha-2-macroglobulin | A2M | 513856 | 0.6266 | 0.0391 |
| Bt_26850_1_S1_at | Bt.26850 | ATP-binding cassette, sub-family G (WHITE), member 4 | ABCG4 | 508443 | 0.7571 | 0.0070 |
| Bt_27396_1_S1_at | Bt.87964 | Abl-interactor 1 | ABI1 | 540815 | 0.6242 | 0.0401 |
| Bt_20171_1_S1_at | Bt.87957 | acyl-Coenzyme A binding domain containing 5 | ACBD5 | 353160 | 0.8827 | 0.0003 |
| Bt_11244_1_S1_at | Bt.11244 | adrenocortical dysplasia homolog (mouse) | ACD | 510353 | -0.6292 | 0.0381 |
| Bt_16790_2_S1_at | Bt.13633 | actinin, alpha 4 | ACTN4 | 522269 | 0.6686 | 0.0245 |
| Bt_734_1_S1_at | Bt.734 | ADAM metallopeptidase domain 9 (meltrin gamma) | ADAM9 | 281600 | 0.6097 | 0.0464 |
| Bt_4496_1_S1_at | Bt.76442 | adenosine A1 receptor | ADORA1 | 282133 | 0.6218 | 0.0411 |
| Bt_9239_1_S1_at | Bt.88336 | adenosine A2b receptor | ADORA2B | 529760 | 0.6688 | 0.0244 |
| Bt_318_1_S1_at | Bt.318 | adrenergic, beta-3-, receptor | ADRB3 | 281606 | 0.8236 | 0.0018 |
| Bt_16782_2_S1_at | Bt.16782 | adenylosuccinate synthase like 1 | ADSSL1 | 784089 | -0.6200 | 0.0419 |
| Bt_9288_2_A1_at | Bt.9288 | actin filament associated protein 1-like 1 | AFAP1L1 | 510738 | -0.6983 | 0.0169 |
| Bt_3115_1_A1_at | Bt.53989 | Aspartylglucosaminidase | AGA | 511345 | -0.6938 | 0.0179 |
| Bt_23626_1_S1_at | Bt.23626 | 1-acylglycerol-3-phosphate O-acyltransferase 5 (lysophosphatidic acid acyltransferase, epsilon) | AGPAT5 | 530414 | -0.7302 | 0.0107 |
| Bt_15900_1_A1_at | Bt.91413 | adenosylhomocysteinase-like 2 | AHCYL2 | 532836 | -0.8339 | 0.0014 |
| Bt_25112_1_S1_at | Bt.57995 | Androgen-induced 1 | AIG1 | 614409 | -0.7208 | 0.0123 |
| Bt_28839_1_S1_at | Bt.28839 | absent in melanoma 1-like | AIM1L | 507142 | -0.6387 | 0.0344 |
| Bt_25210_1_A1_at | Bt.14084 | A kinase (PRKA) anchor protein 8 | AKAP8 | 522905 | -0.7135 | 0.0137 |
| Bt_23094_3_S1_at | Bt.67184 | aldo-keto reductase family 1, member C4 (chlordecone reductase; 3-alpha hydroxysteroid dehydrogenase, type I; dihydrodiol dehydrogenase 4) | AKR1C4 | 282138 | -0.6510 | 0.0301 |
| Bt_3607_1_S1_at | Bt.49467 | aminolevulinate, delta-, synthase 2 | ALAS2 | 511791 | -0.7157 | 0.0133 |
| Bt_436_1_S1_at | Bt.98765 | aldehyde dehydrogenase 6 family, member A1 | ALDH6A1 | 327692 | 0.7212 | 0.0123 |
| Bt_22497_2_S1_at | Bt.65140 | alkB, alkylation repair homolog 2 (E. coli) | ALKBH2 | 511380 | -0.6911 | 0.0185 |
| Bt_8917_1_S1_at | Bt.56010 | arachidonate lipoxygenase, epidermal | ALOX12E | 787450 | -0.7622 | 0.0064 |
| Bt_22046_1_S1_at | Bt.22046 | alpha-methylacyl-CoA racemase | AMACR | 540376 | -0.7171 | 0.0130 |
| Bt_20890_1_S1_at | Bt.20890 | amylase, alpha 2A (pancreatic) | AMY2A | 505049 | -0.6959 | 0.0174 |
| Bt_4323_2_S1_at | Bt.49063 | Archaelysin family metallopeptidase 2 | AMZ2 | 515126 | 0.6040 | 0.0491 |
| Bt_14043_3_S1_at | Bt.91133 | Ankyrin repeat domain 10 | ANKRD10 | 510084 | -0.6865 | 0.0197 |
| Bt_25945_2_A1_a_at | Bt.25945 | ankyrin repeat domain 16 | ANKRD16 | 507779 /// 784882 | -0.7111 | 0.0141 |
| Bt_20493_1_S1_at | Bt.37776 | ankyrin repeat domain 6 | ANKRD6 | 516065 | -0.6880 | 0.0193 |
| Bt_28174_1_S1_a_at | Bt.28174 | annexin A10 | ANXA10 | 505322 | -0.7441 | 0.0086 |
| Bt_2056_1_S1_at | Bt.2056 | N-acylaminoacyl-peptide hydrolase | APEH | 514666 | -0.6385 | 0.0345 |
| Bt_22694_1_A1_at | Bt.22694 | apolipoprotein A-V | APOA5 | 538914 | -0.6460 | 0.0318 |
| Bt_19980_1_A1_at | Bt.56771 | ovarian and testicular apolipoprotein N | ApoN | 445463 | -0.6617 | 0.0266 |
| Bt_16472_1_S1_at | Bt.16472 | aprataxin | APTX | 359714 | -0.8200 | 0.0020 |
| Bt_927_1_A1_at | Bt.927 | ADP-ribosylation factor 1 | ARF1 | 338058 | -0.6643 | 0.0258 |
| Bt_14681_1_A1_at | Bt.97117 | arginase, liver | ARG1 | 513608 | -0.7678 | 0.0058 |
| Bt_24416_2_A1_at | Bt.24416 | Rho GDP dissociation inhibitor (GDI) gamma | ARHGDIG | 613745 | -0.7498 | 0.0079 |
| Bt_27352_2_S1_a_at | Bt.27352 | Rho guanine exchange factor (GEF) 16 | ARHGEF16 | 514453 | -0.8096 | 0.0025 |
| Bt_8133_1_S1_at | Bt.87256 | aryl hydrocarbon receptor nuclear translocator | ARNT | 281010 | 0.6252 | 0.0397 |
| Bt_4823_1_S1_at | Bt.4823 | cAMP-regulated phosphoprotein, 19kDa | ARPP19 | 282658 | 0.6743 | 0.0229 |
| Bt_3227_1_S1_at | Bt.3227 | activating signal cointegrator 1 complex subunit 2 | ASCC2 | 505503 | -0.6193 | 0.0422 |
| Bt_18455_1_A1_at | --- | additional sex combs like 2 (Drosophila) | ASXL2 | 100140419 | 0.7692 | 0.0057 |
| Bt_19161_1_S1_at | Bt.49442 | activating transcription factor 4 (tax-responsive enhancer element B67) | ATF4 | 509107 | 0.8468 | 0.0010 |
| Bt_27780_1_A1_at | Bt.73299 | Atlastin 3 | ATL3 | 515124 | -0.6812 | 0.0210 |
| Bt_29000_1_S1_at | Bt.29000 | atonal homolog 1 (Drosophila) | ATOH1 | 539158 | -0.6251 | 0.0397 |
| Bt_23988_1_A1_at | Bt.10174 | ATPase, H+ transporting, lysosomal 38kDa, V0 subunit d2 | ATP6V0D2 | 511839 | -0.7063 | 0.0151 |
| Bt_21662_2_S1_at | Bt.21662 | ATPase, H+ transporting V0 subunit e2 | ATP6V0E2 | 505224 | 0.6241 | 0.0401 |
| Bt_19311_1_A1_at | Bt.26471 | antizyme inhibitor 1 | AZIN1 | 532835 | -0.6928 | 0.0181 |
| Bt_1272_1_S1_at | Bt.96844 | UDP-GlcNAc:betaGal beta-1,3-N-acetylglucosaminyltransferase 3 | B3GNT3 | 784997 | -0.6238 | 0.0403 |
| Bt_6581_1_S1_at | Bt.6581 | BAH domain and coiled-coil containing 1 | BAHCC1 | 786025 | -0.6789 | 0.0216 |
| Bt_23547_2_S1_at | Bt.23547 | HLA-B associated transcript 3 | BAT3 | 508605 | -0.6205 | 0.0416 |
| Bt_9265_1_A1_at | Bt.9265 | basic leucine zipper transcription factor, ATF-like | BATF | 617628 | 0.6115 | 0.0456 |
| Bt_22524_2_A1_at | Bt.18407 | Bardet-Biedl syndrome 5 | BBS5 | 528191 | -0.7432 | 0.0088 |
| Bt_7542_1_S1_at | Bt.11884 | BCL2-related protein A1 | BCL2A1 | 282151 | 0.6104 | 0.0461 |
| Bt_15605_1_S1_at | Bt.15605 | Beta-carotene 15,15'-monooxygenase 1 | BCMO1 | 534696 | -0.7200 | 0.0125 |
| Bt_18938_1_A1_at | Bt.88847 | BCL6 co-repressor | BCOR | 784529 | -0.7440 | 0.0087 |
| Bt_7220_1_S1_at | Bt.350 | MHC class II antigen | BLA-DQB | 539241 | 0.7736 | 0.0052 |
| Bt_6958_1_A1_at | Bt.1411 | BCL2/adenovirus E1B 19kDa interacting protein 3 | BNIP3 | 615342 | -0.7560 | 0.0071 |
| Bt_25949_1_A1_at | Bt.35142 | Bone morphogenetic protein/retinoic acid inducible neural-specific 1 | BRINP1 | 538990 | -0.8958 | 0.0002 |
| Bt_576_1_S1_at | Bt.576 | common salivary protein BSP30, form b | BSP30B | 286881 | -0.7062 | 0.0152 |
| Bt_1213_1_S1_at | Bt.48374 | BTB (POZ) domain containing 1 | BTBD1 | 525916 | -0.6406 | 0.0337 |
| Bt_12872_1_S1_at | Bt.52740 | BTB (POZ) domain containing 3 | BTBD3 | 513420 | -0.8064 | 0.0027 |
| Bt_29690_1_A1_at | Bt.29690 | T cell receptor delta chain variable region | BVd1.19 | 407203 | -0.6197 | 0.0420 |
| Bt_29688_1_A1_at | Bt.89142 | T cell receptor delta chain variable region | BVd1.22 | 407201 | -0.6061 | 0.0481 |
| Bt_28446_1_A1_at | Bt.28446 | T cell receptor delta chain variable region | BVd1.25 | 407200 | -0.6045 | 0.0488 |
| Bt_28398_1_S1_at | Bt.48891 | brix domain containing 2 | BXDC2 | 518501 | -0.6571 | 0.0280 |
| Bt_10900_1_S1_at | Bt.10900 | chromosome 14 open reading frame 169 ortholog | C10H14ORF169 | 511031 | -0.6097 | 0.0464 |
| Bt_26211_1_S1_at | Bt.17838 | Chromosome 10 open reading frame 107 | C10orf107 | 510670 | -0.6037 | 0.0492 |
| Bt_17066_1_A1_at | Bt.17066 | chromosome 9 open reading frame 116 ortholog | C11H9ORF116 | 618654 | -0.7232 | 0.0119 |
| Bt_21720_1_S1_at | Bt.49503 | chromosome 11 open reading frame 74 ortholog | C15H11orf74 | 614192 | -0.7685 | 0.0057 |
| Bt_17916_1_A1_at | Bt.39171 | Chromosome 21 open reading frame 91 ortholog | C1H21ORF91 | 540784 | -0.6081 | 0.0472 |
| Bt_10058_1_S1_at | Bt.102353 | Chromosome 14 open reading frame 129 ortholog | C21H14ORF129 | 616052 | -0.6559 | 0.0284 |
| Bt_25289_1_A1_at | Bt.49501 | Chromosome 15 open reading frame 40 ortholog | C21H15orf40 | 509050 | -0.7270 | 0.0112 |
| Bt_2481_2_S1_at | Bt.23872 | Chromosome 6 open reading frame 105 ortholog | C23H6ORF105 | 613714 | -0.7399 | 0.0092 |
| Bt_21401_2_S1_at | Bt.14645 | Chromosome 6 open reading frame 206 ortholog | C23H6orf206 | 523327 | -0.6385 | 0.0345 |
| Bt_22485_1_S1_at | Bt.22485 | chromosome 16 open reading frame 53 ortholog | C25H16orf53 | 513650 | -0.6191 | 0.0423 |
| Bt_22612_1_A1_at | Bt.22612 | chromosome 8 open reading frame 40 ortholog | C27H8orf40 | 515895 | -0.6073 | 0.0475 |
| Bt_4209_1_S1_a_at | Bt.19562 | complement component 3 | C3 | 280677 | 0.7276 | 0.0112 |
| Bt_19664_1_A1_at | Bt.42408 | chromosome 1 open reading frame 210 ortholog | C3H1ORF210 | 509100 | -0.6347 | 0.0359 |
| Bt_24280_1_A1_at | Bt.24280 | chromosome 1 open reading frame 41 ortholog | C3H1ORF41 | 616194 | -0.7781 | 0.0048 |
| Bt_22375_2_A1_at | Bt.46083 | chromosome 1 open reading frame 66 ortholog | C3H1orf66 | 532204 | 0.6578 | 0.0278 |
| Bt_10018_2_A1_at | Bt.49072 | Chromosome 5 open reading frame 15 ortholog | C7H5ORF15 | 514781 | -0.8091 | 0.0026 |
| Bt_11073_1_S1_at | Bt.21487 | chromosome 9 open reading frame 21 ortholog | C8H9orf21 | 616897 | -0.6738 | 0.0230 |
| Bt_12607_1_A1_at | Bt.12607 | chromosome 6 open reading frame 185 ortholog | C9H6orf185 | 515167 | -0.6556 | 0.0285 |
| Bt_13845_1_S1_at | Bt.13845 | chromosome 6 open reading frame 203 ortholog | C9H6ORF203 | 613695 | -0.8683 | 0.0005 |
| Bt_17479_1_A1_at | Bt.55324 | calcium binding tyrosine-(Y)-phosphorylation regulated | CABYR | 510319 | -0.7378 | 0.0095 |
| Bt_2617_1_S1_at | Bt.2617 | calpain 10 | CAPN10 | 789674 | -0.7752 | 0.0051 |
| Bt_3961_2_S1_a_at | Bt.3961 | calpain 3, (p94) | CAPN3 | 281663 | -0.6327 | 0.0367 |
| Bt_26083_1_A1_at | Bt.26083 | Calpain 5 | CAPN5 | 536988 | -0.6037 | 0.0492 |
| Bt_20860_1_A1_at | Bt.20860 | calcyphosine-like | CAPSL | 507306 | -0.6380 | 0.0347 |
| Bt_687_1_S1_at | Bt.43438 | caspase recruitment domain family, member 11 | CARD11 | 515377 | 0.6414 | 0.0334 |
| Bt_20565_1_A1_at | Bt.20565 | cancer susceptibility candidate 4 | CASC4 | 540780 | 0.6204 | 0.0417 |
| Bt_22877_1_S1_at | Bt.22877 | catecholamine binding protein CBP40 | CBP40 | 353122 | -0.7283 | 0.0110 |
| Bt_7872_1_S1_at | Bt.7872 | Hypothetical LOC780962 | CBR1 | 515946 | -0.6768 | 0.0222 |
| Bt_21034_2_S1_at | Bt.21034 | coiled-coil and C2 domain containing 1B | CC2D1B | 511350 | -0.7148 | 0.0134 |
| Bt_27498_1_S1_at | Bt.3499 | Coiled-coil domain containing 104 | CCDC104 | 513777 | -0.6405 | 0.0338 |
| Bt_27098_1_A1_at | Bt.55869 | coiled-coil domain containing 37 | CCDC37 | 519687 | -0.7348 | 0.0100 |
| Bt_11029_1_S1_at | Bt.11029 | Coiled-coil domain containing 8 | CCDC8 | 616838 | -0.6475 | 0.0312 |
| Bt_45_1_S1_at | Bt.45 | calicin | CCIN | 281041 | -0.6301 | 0.0377 |
| Bt_25697_1_A1_a_at | Bt.25697 | chemokine (C-C motif) receptor 9 | CCR9 | 530951 | -0.8120 | 0.0024 |
| Bt_25985_1_A1_at | Bt.67940 | CD163 molecule-like 1 /// similar to BoWC1.1 /// similar to BoWC1.1 /// WC1 isolate CH218 /// WC1 isolate DV9 /// similar to BoWC1.1 /// similar to BoWC1.1 /// BoWC1.1 | CD163L1 /// LOC522910 /// LOC523576 /// LOC751805 /// LOC751809 /// LOC785733 /// LOC786090 /// WC1 | 338056 /// 522910 /// 523576 /// 751805 /// 751809 /// 783109 /// 785733 /// 786090 | -0.6561 | 0.0284 |
| Bt_24979_1_S1_at | Bt.49600 | CD1e molecule | CD1E | 510832 | -0.7291 | 0.0109 |
| Bt_18756_1_S1_at | Bt.7917 | CDC42 small effector 2 | CDC42SE2 | 789618 | 0.6411 | 0.0335 |
| Bt_2696_2_S1_at | Bt.81912 | cadherin 11, type 2, OB-cadherin (osteoblast) /// similar to Cadherin-11 precursor (Osteoblast cadherin) (OB-cadherin) (OSF-4) | CDH11 /// LOC790083 | 785475 /// 788509 /// 790083 | -0.6050 | 0.0486 |
| Bt_5050_1_A1_at | Bt.12967 | cadherin 16, KSP-cadherin | CDH16 | 508777 | -0.8096 | 0.0025 |
| Bt_22785_1_S1_at | Bt.64509 | CCAAT/enhancer binding protein (C/EBP), beta | CEBPB | 338319 | 0.6230 | 0.0406 |
| Bt_6406_2_A1_at | Bt.6406 | CCAAT/enhancer binding protein (C/EBP), delta | CEBPD | 281678 | 0.7713 | 0.0054 |
| Bt_23571_2_A1_at | Bt.65870 | centrosomal protein 350kDa | CEP350 | 534896 | 0.6882 | 0.0192 |
| Bt_18643_2_S1_at | Bt.29565 | CASP8 and FADD-like apoptosis regulator | CFLAR | 497199 | 0.7009 | 0.0163 |
| Bt_5238_1_S1_at | Bt.60417 | chitinase 3-like 1 (cartilage glycoprotein-39) | CHI3L1 | 286869 | 0.6051 | 0.0486 |
| Bt_13038_1_A1_at | Bt.13038 | cholinergic receptor, nicotinic, beta 2 (neuronal) | CHRNB2 | 517519 | -0.7549 | 0.0072 |
| Bt_10303_1_S1_at | Bt.10303 | carbohydrate (N-acetylgalactosamine 4-0) sulfotransferase 14 | CHST14 | 511245 | -0.6352 | 0.0357 |
| Bt_21607_1_S1_at | Bt.21607 | Cbp/p300-interacting transactivator, with Glu/Asp-rich carboxy-terminal domain, 2 | CITED2 | 521378 | 0.6218 | 0.0411 |
| Bt_3885_1_S1_at | Bt.3885 | chloride channel accessory 3 (pseudogene) | CLCA3P | 281694 | 0.7430 | 0.0088 |
| Bt_22180_1_S1_at | Bt.22180 | C-type lectin domain family 4, member G | CLEC4G | 507332 | -0.7213 | 0.0122 |
| Bt_20954_1_S1_at | Bt.20954 | clusterin associated protein 1 | CLUAP1 | 617589 | -0.6184 | 0.0425 |
| Bt_21931_1_S1_at | Bt.21931 | CKLF-like MARVEL transmembrane domain containing 7 | CMTM7 | 532269 | 0.6715 | 0.0237 |
| Bt_27901_1_S1_at | --- | CNKSR family member 3 | CNKSR3 | 783484 | -0.6332 | 0.0365 |
| Bt_10004_1_A1_at | Bt.46623 | ciliary neurotrophic factor receptor | CNTFR | 539548 | 0.6683 | 0.0246 |
| Bt_21409_1_A1_at | Bt.9656 | Collagen, type XI, alpha 1 | COL11A1 | 287013 | -0.6197 | 0.0420 |
| Bt_24550_1_S1_a_at | Bt.24550 | catechol-O-methyltransferase domain containing 1 | COMTD1 | 514949 | -0.6227 | 0.0407 |
| Bt_26836_1_A1_at | Bt.87535 | Coenzyme Q2 homolog, prenyltransferase (yeast) | COQ2 | 504633 | -0.8209 | 0.0020 |
| Bt_17910_2_S1_at | Bt.28800 | coronin, actin binding protein, 2A | CORO2A | 617620 | -0.6583 | 0.0276 |
| Bt_892_1_S1_at | Bt.49046 | cytochrome c oxidase subunit VIIa polypeptide 2 like | COX7A2L | 540225 | 0.6235 | 0.0404 |
| Bt_22390_3_A1_at | Bt.76429 | ceruloplasmin (ferroxidase) | CP | 514194 | 0.6282 | 0.0385 |
| Bt_19969_1_S1_a_at | Bt.46077 | carboxypeptidase A1 (pancreatic) /// carboxypeptidase A4 /// carboxypeptidase A5 | CPA1 /// CPA4 /// CPA5 | 286762 /// 511416 /// 512903 | -0.6569 | 0.0281 |
| Bt_20134_2_S1_at | Bt.62807 | carboxypeptidase N, polypeptide 1 | CPN1 | 536753 | -0.6547 | 0.0288 |
| Bt_22472_1_S1_at | Bt.48141 | CAMP responsive element modulator | CREM | 540605 | 0.6961 | 0.0173 |
| Bt_22950_1_A1_at | --- | cysteine-rich secretory protein 1 | CRISP-1 | 353291 | -0.7725 | 0.0053 |
| Bt_18188_1_A1_at | Bt.55503 | cysteine-rich secretory protein LCCL domain containing 2 | CRISPLD2 | 505329 | -0.8596 | 0.0007 |
| Bt_21479_1_A1_at | Bt.102121 | Crooked neck pre-mRNA splicing factor-like 1 (Drosophila) | CRNKL1 | 526536 | 0.6658 | 0.0253 |
| Bt_4899_2_S1_at | Bt.44393 | CREB regulated transcription coactivator 1 | CRTC1 | 510465 | 0.7475 | 0.0082 |
| Bt_8995_1_A1_at | Bt.8995 | cryptochrome 1 (photolyase-like) | CRY1 | 535947 | -0.7823 | 0.0044 |
| Bt_382_1_S1_at | Bt.382 | crystallin, beta A1 | CRYBA1 | 282202 | -0.6986 | 0.0168 |
| Bt_598_1_A1_at | Bt.598 | crystallin, beta B3 | CRYBB3 | 282206 | -0.7072 | 0.0149 |
| Bt_13518_2_S1_at | Bt.31290 | crystallin, zeta (quinone reductase)-like 1 | CRYZL1 | 506426 | -0.6082 | 0.0471 |
| Bt_10184_1_S1_a_at | Bt.46945 | casein kinase 1, delta | CSNK1D | 407122 /// 523542 | 0.6265 | 0.0392 |
| Bt_13321_1_S1_at | Bt.13321 | centrosome and spindle pole associated protein 1 | CSPP1 | 524180 | -0.6100 | 0.0463 |
| Bt_7165_1_S1_at | Bt.7165 | chemokine (C-X-C motif) ligand 5 | CXCL5 | 281735 | 0.6594 | 0.0273 |
| Bt_20035_1_S1_at | Bt.9593 | CXXC finger 5 | CXXC5 | 538485 | -0.6021 | 0.0500 |
| Bt_22938_1_S1_at | Bt.50017 | cylicin, basic protein of sperm head cytoskeleton 2 | CYLC2 | 281738 | -0.6491 | 0.0307 |
| Bt_27817_1_S1_at | Bt.58637 | cylindromatosis (turban tumor syndrome) | CYLD | 536421 | 0.6628 | 0.0262 |
| Bt_23912_1_A1_a_at | Bt.5532 | Cytochrome P450 subfamily IIE polypeptide 1 | CYP2E1 | 282213 | 0.6310 | 0.0373 |
| Bt_4126_1_A1_at | Bt.91691 | cytochrome P450, family 4, subfamily A, polypeptide 11 | CYP4A11 | 511890 | 0.6155 | 0.0438 |
| Bt_15382_1_A1_at | Bt.15382 | Disabled homolog 2, mitogen-responsive phosphoprotein (Drosophila) | DAB2 | 509221 | -0.6141 | 0.0444 |
| Bt_3726_1_A1_at | Bt.3726 | Development and differentiation enhancing factor 2 | DDEF2 | 618009 | -0.6206 | 0.0416 |
| Bt_7540_3_S1_at | Bt.46278 | discoidin domain receptor tyrosine kinase 1 | DDR1 | 534092 | -0.6171 | 0.0431 |
| Bt_13425_1_A1_at | Bt.13425 | DEAD (Asp-Glu-Ala-Asp) box polypeptide 20 | DDX20 | 528433 | -0.6117 | 0.0455 |
| Bt_132_1_S1_at | Bt.132 | defensin, beta 1 | DEFB1 | 281743 | 0.6630 | 0.0262 |
| Bt_13125_1_S1_at | Bt.13125 | defensin, beta 1 /// defensin, beta 5 | DEFB1 /// DEFB5 | 281743 /// 783935 | 0.7262 | 0.0114 |
| Bt_29930_1_S1_at | Bt.63223 | DENN/MADD domain containing 3 | DENND3 | 508078 | 0.7117 | 0.0140 |
| Bt_21056_1_S1_at | Bt.21056 | Der1-like domain family, member 3 | DERL3 | 614334 | 0.6578 | 0.0278 |
| Bt_26727_1_S1_at | Bt.87215 | dehydrodolichyl diphosphate synthase | DHDDS | 523264 | 0.6103 | 0.0462 |
| Bt_27335_1_A1_at | Bt.27335 | Dehydrogenase/reductase (SDR family) member 13 | DHRS13 | 514903 | -0.7173 | 0.0130 |
| Bt_16532_1_A1_at | Bt.42016 | DEAH (Asp-Glu-Ala-His) box polypeptide 34 | DHX34 | 506965 | -0.6354 | 0.0356 |
| Bt_12552_1_S1_at | Bt.52291 | DEAH (Asp-Glu-Ala-His) box polypeptide 35 | DHX35 | 513148 | -0.6081 | 0.0471 |
| Bt_4467_2_S1_a_at | Bt.23365 | diablo homolog (Drosophila) | DIABLO | 493999 | -0.6046 | 0.0488 |
| Bt_28463_1_S1_at | Bt.45343 | dicer 1, ribonuclease type III | DICER1 | 337871 | -0.6128 | 0.0450 |
| Bt_13880_1_S1_at | Bt.13880 | Dickkopf homolog 1 (Xenopus laevis) | DKK1 | 504445 | 0.6569 | 0.0281 |
| Bt_23055_1_A1_s_at | Bt.11856 | discs, large homolog 5 (Drosophila) | DLG5 | 535699 | -0.6676 | 0.0248 |
| Bt_19640_1_A1_at | Bt.31618 | DMRT-like family A2 | DMRTA2 | 537680 | -0.6150 | 0.0440 |
| Bt_26805_1_S1_at | Bt.18516 | DnaJ (Hsp40) homolog, subfamily A, member 4 | DNAJA4 | 528615 | 0.6238 | 0.0402 |
| Bt_26818_1_S1_at | Bt.26818 | DnaJ (Hsp40) homolog, subfamily B, member 9 | DNAJB9 | 614588 | 0.6483 | 0.0310 |
| Bt_26495_1_S1_at | Bt.59126 | DnaJ (Hsp40) homolog, subfamily C, member 19 | DNAJC19 | 513918 | -0.6207 | 0.0416 |
| Bt_1346_1_S1_at | Bt.64853 | DnaJ (Hsp40) homolog, subfamily C, member 5 | DNAJC5 | 282216 | -0.7269 | 0.0113 |
| Bt_10620_1_S1_at | Bt.30953 | dedicator of cytokinesis 1 | DOCK1 | 537203 | -0.7538 | 0.0074 |
| Bt_24916_1_A1_at | Bt.53434 | dedicator of cytokinesis 7 | DOCK7 | 537464 | -0.6218 | 0.0411 |
| Bt_6145_1_S1_at | Bt.61023 | DPH3, KTI11 homolog (S. cerevisiae) | DPH3 | 511579 | 0.7242 | 0.0117 |
| Bt_26282_1_A1_at | Bt.26282 | dopamine receptor D5 | DRD5 | 526221 | -0.6874 | 0.0194 |
| Bt_25705_1_A1_at | Bt.21578 | Dual specificity phosphatase 23 | DUSP23 | 513978 | -0.6204 | 0.0417 |
| Bt_17972_1_A1_at | Bt.17972 | dishevelled, dsh homolog 2 (Drosophila) | DVL2 | 614312 | -0.6845 | 0.0201 |
| Bt_22186_1_A1_at | Bt.22186 | DPY30 domain containing 2 | DYDC2 | 514060 | -0.6393 | 0.0342 |
| Bt_10818_1_A1_at | Bt.10818 | Dynein, cytoplasmic 2, light intermediate chain 1 | DYNC2LI1 | 507311 | -0.6389 | 0.0343 |
| Bt_20827_1_A1_at | Bt.18339 | dynein, light chain, roadblock-type 2 | DYNLRB2 | 768062 | -0.6547 | 0.0288 |
| Bt_19304_1_A1_at | Bt.19304 | DAZ interacting protein 1 | DZIP1 | 506546 | -0.6428 | 0.0329 |
| Bt_5885_1_S1_at | Bt.5885 | enoyl Coenzyme A hydratase domain containing 2 | ECHDC2 | 513795 | -0.6740 | 0.0229 |
| Bt_25506_1_A1_at | Bt.25506 | EF-hand calcium binding domain 1 | EFCAB1 | 505272 | -0.7022 | 0.0160 |
| Bt_27883_1_S1_at | Bt.23386 | embryonal Fyn-associated substrate | EFS | 508559 | -0.6096 | 0.0465 |
| Bt_2096_2_S1_at | Bt.2096 | EH-domain containing 4 | EHD4 | 505206 | 0.6418 | 0.0333 |
| Bt_26583_2_S1_at | Bt.2975 | Euchromatic histone-lysine N-methyltransferase 1 | EHMT1 | 528606 | -0.6182 | 0.0427 |
| Bt_19313_1_A1_at | Bt.10192 | Eukaryotic translation initiation factor 2B, subunit 3 gamma, 58kDa | EIF2B3 | 534063 | -0.6072 | 0.0476 |
| Bt_13828_1_S1_a_at | Bt.13828 | engulfment and cell motility 3 | ELMO3 | 525427 | -0.6824 | 0.0207 |
| Bt_1205_1_A1_at | Bt.1205 | elongation protein 2 homolog (S. cerevisiae) | ELP2 | 511041 | -0.6964 | 0.0173 |
| Bt_20101_2_S1_a_at | Bt.20101 | echinoderm microtubule associated protein like 3 | EML3 | 504884 | 0.6165 | 0.0434 |
| Bt_6206_1_S1_at | Bt.6206 | endo-beta-N-acetylglucosaminidase | ENGASE | 526608 | -0.6068 | 0.0477 |
| Bt_25979_1_S1_a_at | Bt.29990 | ectonucleoside triphosphate diphosphohydrolase 4 | ENTPD4 | 531411 | 0.7409 | 0.0091 |
| Bt_3718_1_S1_at | Bt.54429 | ectonucleoside triphosphate diphosphohydrolase 6 (putative function) | ENTPD6 | 508097 | -0.7892 | 0.0039 |
| Bt_4353_2_S1_at | Bt.45570 | endothelial PAS domain protein 1 | EPAS1 | 282711 | 0.6906 | 0.0186 |
| Bt_25447_1_A1_at | Bt.61672 | EPH receptor A7 | EPHA7 | 538797 | -0.6188 | 0.0424 |
| Bt_599_1_S1_at | Bt.65453 | epiphycan | EPYC | 281747 | -0.7141 | 0.0136 |
| Bt_1078_1_A1_at | Bt.102211 | epithelial splicing regulatory protein 2 | ESRP2 | 510878 | -0.6428 | 0.0329 |
| Bt_11114_1_S1_at | Bt.11114 | exocyst complex component 3 | EXOC3 | 513138 | -0.6702 | 0.0240 |
| Bt_1345_1_S1_at | Bt.24447 | coagulation factor II (thrombin) receptor-like 2 | F2RL2 | 512581 | 0.7211 | 0.0123 |
| Bt_4106_1_S1_at | Bt.4106 | coagulation factor III (thromboplastin, tissue factor) | F3 | 280686 /// 784008 | 0.8018 | 0.0030 |
| Bt_3682_1_S1_at | Bt.88785 | Fas (TNFRSF6)-associated via death domain | FADD | 493720 | 0.6587 | 0.0275 |
| Bt_836_1_S1_at | Bt.88636 | fumarylacetoacetate hydrolase domain containing 1 | FAHD1 | 509273 | -0.7016 | 0.0161 |
| Bt_16370_1_S1_at | Bt.87967 | family with sequence similarity 107, member B | FAM107B | 535023 | 0.6718 | 0.0236 |
| Bt_27314_2_S1_at | Bt.27314 | family with sequence similarity 108, member B1 | FAM108B1 | 781153 | -0.6150 | 0.0441 |
| Bt_20247_1_S1_at | Bt.59267 | family with sequence similarity 117, member A | FAM117A | 509931 | -0.6400 | 0.0339 |
| Bt_19613_1_S1_at | Bt.19613 | family with sequence similarity 3, member C | FAM3C | 615690 | 0.6679 | 0.0247 |
| Bt_1066_3_S1_a_at | Bt.49606 | Family with sequence similarity 50, member A | FAM50A | 515539 | 0.7183 | 0.0128 |
| Bt_18725_1_A1_at | Bt.18725 | Family with sequence similarity 81, member B | FAM81B | 514910 | -0.6703 | 0.0240 |
| Bt_27103_1_A1_at | Bt.27103 | FAST kinase domains 1 | FASTKD1 | 515744 | -0.6492 | 0.0307 |
| Bt_7257_1_S1_at | --- | FAST kinase domains 5 | FASTKD5 | 788680 | -0.7660 | 0.0060 |
| Bt_20361_1_S1_at | Bt.20361 | F-box and leucine-rich repeat protein 20 | FBXL20 | 511007 | 0.7500 | 0.0079 |
| Bt_21681_2_S1_at | Bt.21681 | F-box and leucine-rich repeat protein 4 | FBXL4 | 535452 | -0.6162 | 0.0435 |
| Bt_18548_1_A1_at | Bt.11397 | F-box protein 11 | FBXO11 | 508172 | 0.7696 | 0.0056 |
| Bt_13844_2_S1_at | Bt.13844 | F-box protein 33 | FBXO33 | 539998 | 0.7789 | 0.0047 |
| Bt_28585_1_A1_at | Bt.28585 | F-box protein 36 | FBXO36 | 617339 | -0.6552 | 0.0286 |
| Bt_20480_1_S1_at | Bt.43176 | F-box and WD repeat domain containing 9 | FBXW9 | 532538 | -0.6253 | 0.0396 |
| Bt_21724_3_A1_at | Bt.14255 | Farnesyl-diphosphate farnesyltransferase 1 | FDFT1 | 281767 | -0.7084 | 0.0147 |
| Bt_23853_3_S1_at | Bt.53298 | Feline sarcoma oncogene | FES | 507304 | 0.6451 | 0.0321 |
| Bt_25548_1_A1_at | Bt.47633 | FEZ family zinc finger 1 | FEZF1 | 511770 | -0.6265 | 0.0391 |
| Bt_13204_1_S1_at | Bt.13204 | fibrinogen-like 1 | FGL1 | 508090 | -0.6163 | 0.0435 |
| Bt_19999_2_S1_at | Bt.19999 | FIC domain containing | FICD | 505923 | 0.7790 | 0.0047 |
| Bt_8158_1_S1_at | Bt.66213 | Forkhead box G1 | FOXG1 | 516947 | -0.8943 | 0.0002 |
| Bt_6180_2_S1_at | Bt.89958 | FSHD region gene 1 | FRG1 | 512533 | 0.7631 | 0.0063 |
| Bt_22221_1_S1_at | Bt.31385 | Fibronectin type III and SPRY domain containing 2 | FSD2 | 540530 | -0.6089 | 0.0468 |
| Bt_405_1_S1_at | Bt.405 | follistatin | FST | 327681 | -0.6664 | 0.0251 |
| Bt_2190_1_S1_at | Bt.2190 | far upstream element (FUSE) binding protein 3 | FUBP3 | 541080 | -0.6194 | 0.0421 |
| Bt_17029_1_A1_at | Bt.17029 | Frataxin | FXN | 505694 | -0.6660 | 0.0253 |
| Bt_20096_2_S1_a_at | Bt.20096 | FXYD domain containing ion transport regulator 5 | FXYD5 | 505584 | 0.6622 | 0.0264 |
| Bt_2582_1_S1_at | Bt.65693 | UDP-N-acetyl-alpha-D-galactosamine:polypeptide N-acetylgalactosaminyltransferase 1 (GalNAc-T1) | GALNT1 | 282241 | 0.6087 | 0.0469 |
| Bt_1484_1_S1_at | Bt.53822 | UDP-N-acetyl-alpha-D-galactosamine:polypeptide N-acetylgalactosaminyltransferase 2 (GalNAc-T2) | GALNT2 | 616218 | -0.6550 | 0.0287 |
| Bt_312_1_S1_at | Bt.312 | gastrin | GAST | 280800 | -0.7239 | 0.0118 |
| Bt_5527_2_S1_a_at | Bt.5527 | glutamyl-tRNA(Gln) amidotransferase, subunit C homolog (bacterial) | GATC | 615845 | -0.7465 | 0.0083 |
| Bt_16037_1_S1_at | Bt.16037 | Globoside alpha-1,3-N-acetylgalactosaminyltransferase 1 | GBGT1 | 783982 | -0.6917 | 0.0184 |
| Bt_21773_1_A1_at | Bt.16350 | guanylate binding protein 5 | GBP5 | 516949 | 0.6024 | 0.0498 |
| Bt_15184_1_A1_at | Bt.53584 | group-specific component (vitamin D binding protein) | GC | 530076 | -0.6582 | 0.0277 |
| Bt_26102_1_A1_at | Bt.26102 | Ganglioside induced differentiation associated protein 2 | GDAP2 | 508774 | 0.6921 | 0.0183 |
| Bt_7047_1_S1_at | Bt.7047 | growth differentiation factor 9 | GDF9 | 282574 | -0.6580 | 0.0278 |
| Bt_10996_1_S1_at | Bt.37948 | glycerophosphodiester phosphodiesterase domain containing 2 | GDPD2 | 514673 | -0.6506 | 0.0302 |
| Bt_22128_1_S1_at | Bt.22128 | glucose-fructose oxidoreductase domain containing 2 | GFOD2 | 507361 | -0.7551 | 0.0072 |
| Bt_13777_2_S1_at | Bt.20772 | GTPase, IMAP family member 7 | GIMAP7 | 100125415 | 0.6120 | 0.0454 |
| Bt_25989_1_A1_at | Bt.35935 | Gap junction protein, beta 6, 30kDa | GJB6 | 508454 | -0.6469 | 0.0314 |
| Bt_29019_1_S1_at | Bt.29019 | gastrokine 1 | GKN1 | 407211 | 0.6650 | 0.0256 |
| Bt_1329_1_S1_at | Bt.5511 | Guanine nucleotide binding protein (G protein), alpha 11 (Gq class) | GNA11 | 281788 | -0.6171 | 0.0431 |
| Bt_16798_1_A1_at | Bt.64619 | GNAS complex locus | GNAS | 281793 | -0.6471 | 0.0314 |
| Bt_409_1_S1_at | Bt.66016 | guanine nucleotide binding protein (G protein), gamma 5 | GNG5 | 287018 | 0.6063 | 0.0480 |
| Bt_13448_2_S1_at | Bt.41956 | Glyceronephosphate O-acyltransferase | GNPAT | 538800 | 0.7178 | 0.0129 |
| Bt_13408_2_S1_at | --- | golgin B1, golgi integral membrane protein | GOLGB1 | 532923 | 0.7023 | 0.0160 |
| Bt_29268_1_S1_at | Bt.29268 | golgi transport 1 homolog A (S. cerevisiae) | GOLT1A | 508464 | -0.6577 | 0.0278 |
| Bt_14464_1_A1_at | Bt.53642 | Gephyrin | GPHN | 535194 | -0.6148 | 0.0441 |
| Bt_27053_1_A1_at | --- | G protein-coupled receptor 1 | GPR1 | 509707 | -0.6038 | 0.0492 |
| Bt_20357_2_A1_at | Bt.20357 | G protein-coupled receptor 132 | GPR132 | 539146 | 0.6351 | 0.0358 |
| Bt_9893_1_S1_at | Bt.9893 | G protein-coupled receptor 88 | GPR88 | 781302 | 0.6236 | 0.0404 |
| Bt_18782_1_S1_at | Bt.18782 | G protein-coupled receptor, family C, group 5, member A | GPRC5A | 516026 | 0.6155 | 0.0438 |
| Bt_7575_1_A1_at | Bt.65463 | Glutamic pyruvate transaminase (alanine aminotransferase) 2 | GPT2 | 618400 | -0.6897 | 0.0189 |
| Bt_12916_1_S1_at | Bt.12916 | glutathione peroxidase 3 (plasma) | GPX3 | 281210 | 0.6197 | 0.0420 |
| Bt_24356_1_S1_at | Bt.24356 | growth factor receptor-bound protein 10 | Grb10 | 407210 | 0.7101 | 0.0143 |
| Bt_16580_3_A1_at | Bt.58358 | grainyhead-like 1 (Drosophila) | GRHL1 | 617248 | -0.6608 | 0.0269 |
| Bt_26217_1_A1_at | Bt.59467 | Glutamate receptor interacting protein 2 | GRIP2 | 509537 | -0.7610 | 0.0065 |
| Bt_12496_1_S1_at | Bt.62317 | glucocorticoid receptor DNA binding factor 1 | GRLF1 | 540310 | -0.6357 | 0.0355 |
| Bt_5304_1_S1_at | Bt.5304 | glutathione S-transferase mu 3 (brain) | GSTM3 | 615507 | -0.6266 | 0.0391 |
| Bt_21893_1_S1_at | Bt.21893 | glutathione S-transferase, theta 3 | GSTT3 | 516190 | -0.6952 | 0.0176 |
| Bt_1892_1_S1_at | Bt.37386 | GTP binding protein 1 | GTPBP1 | 513922 | -0.6107 | 0.0460 |
| Bt_4538_1_S1_at | Bt.4538 | guanylate cyclase 2C (heat stable enterotoxin receptor) | GUCY2C | 282244 | -0.6154 | 0.0439 |
| Bt_21366_2_S1_at | Bt.23788 | GUF1 GTPase homolog (S. cerevisiae) | GUF1 | 522459 | -0.6426 | 0.0330 |
| Bt_23607_1_S1_at | Bt.58717 | hydroxyacyl-Coenzyme A dehydrogenase | HADH | 532785 | -0.6252 | 0.0397 |
| Bt_832_1_S1_at | Bt.52915 | hydroxyacyl-Coenzyme A dehydrogenase/3-ketoacyl-Coenzyme A thiolase/enoyl-Coenzyme A hydratase (trifunctional protein), beta subunit | HADHB | 281811 | -0.6528 | 0.0294 |
| Bt_29861_1_A1_at | Bt.63175 | hyaluronan receptor for endocytosis-like | hare | 407177 | -0.7311 | 0.0106 |
| Bt_8263_1_A1_at | Bt.55961 | hect (homologous to the E6-AP (UBE3A) carboxyl terminus) domain and RCC1 (CHC1)-like domain (RLD) 1 | HERC1 | 538029 | 0.7240 | 0.0118 |
| Bt_22003_1_S1_at | Bt.22003 | hect domain and RLD 2 | HERC2 | 535440 | -0.6599 | 0.0271 |
| Bt_20010_1_S1_at | Bt.20010 | Homocysteine-inducible, endoplasmic reticulum stress-inducible, ubiquitin-like domain member 1 | HERPUD1 | 613577 | 0.6608 | 0.0269 |
| Bt_16348_2_S1_at | Bt.16348 | HERPUD family member 2 | HERPUD2 | 512265 | 0.6083 | 0.0470 |
| Bt_18973_1_S1_a_at | Bt.62451 | hexosaminidase (glycosyl hydrolase family 20, catalytic domain) containing | HEXDC | 506184 | -0.6677 | 0.0248 |
| Bt_2337_1_A1_at | Bt.41831 | homeodomain interacting protein kinase 1 | HIPK1 | 512233 | -0.6063 | 0.0480 |
| Bt_24340_1_S1_at | Bt.24340 | HIRA interacting protein 3 | HIRIP3 | 539704 | -0.6064 | 0.0480 |
| Bt_13920_1_S1_at | Bt.13920 | Histone cluster 1, H2bn | HIST1H2BN | 614958 | 0.6473 | 0.0313 |
| Bt_28399_1_S1_at | Bt.8012 | human immunodeficiency virus type I enhancer binding protein 2 | HIVEP2 | 540396 | 0.6188 | 0.0424 |
| Bt_26627_1_S1_at | Bt.41992 | HNF1 homeobox B | HNF1B | 503581 | -0.6158 | 0.0437 |
| Bt_28155_1_S1_at | Bt.59423 | homeobox A4 | HOXA4 | 538865 | -0.7127 | 0.0138 |
| Bt_24731_1_S1_at | Bt.24731 | homeobox D10 | HOXD10 | 540251 | -0.6492 | 0.0307 |
| Bt_27970_1_S1_at | Bt.27970 | Hermansky-Pudlak syndrome 6 | HPS6 | 511792 | -0.8483 | 0.0010 |
| Bt_13027_1_A1_at | Bt.13027 | hydroxysteroid (11-beta) dehydrogenase 1 | HSD11B1 | 282589 | 0.6116 | 0.0456 |
| Bt_26685_1_S1_at | Bt.26685 | heat shock 27kDa protein 3 | HSPB3 | 616007 | -0.7863 | 0.0041 |
| Bt_27989_1_A1_at | Bt.79134 | 5-hydroxytryptamine (serotonin) receptor 2B | HTR2B | 407135 | -0.6038 | 0.0492 |
| Bt_10437_1_S1_at | Bt.98746 | isoleucyl-tRNA synthetase 2, mitochondrial | IARS2 | 533057 | -0.6116 | 0.0456 |
| Bt_17999_3_A1_a_at | Bt.19427 | Intercellular adhesion molecule 2 | ICAM2 | 506088 | 0.8549 | 0.0008 |
| Bt_24712_1_S1_at | Bt.24712 | inducible T-cell co-stimulator | ICOS | 507026 | 0.6248 | 0.0398 |
| Bt_4557_1_S1_at | Bt.4557 | interferon, alpha; receptor | IFNAR1 | 282257 | 0.7122 | 0.0139 |
| Bt_8829_1_S1_a_at | Bt.37431 | Intraflagellar transport 122 homolog (Chlamydomonas) | IFT122 | 536731 | -0.6533 | 0.0293 |
| Bt_26975_1_A1_a_at | Bt.77163 | intraflagellar transport 172 homolog (Chlamydomonas) | IFT172 | 539164 | -0.6058 | 0.0482 |
| Bt_12490_2_A1_x_at | Bt.97040 | IgG2a heavy chain constant region | IgCgamma | 404109 | 0.6316 | 0.0371 |
| Bt_12759_1_A1_at | Bt.12759 | insulin-like growth factor 1 receptor | IGF1R | 281848 | 0.6088 | 0.0468 |
| Bt_5237_1_S1_at | Bt.5237 | insulin-like growth factor binding protein 4 | IGFBP4 | 282262 | 0.6127 | 0.0451 |
| Bt_28447_1_S1_x_at | Bt.12490 | immunoglobulin heavy constant gamma 1 | IGHG1 | 281850 | 0.6906 | 0.0186 |
| Bt_3843_1_S1_at | Bt.101852 | immunoglobulin J chain | IGJ | 280821 | 0.7618 | 0.0064 |
| Bt_12906_2_S1_at | Bt.57604 | Ig kappa chain | IGK | 506890 | 0.6313 | 0.0372 |
| Bt_21368_1_S1_s_at | Bt.95139 | immunoglobulin light chain, lambda gene cluster /// immunoglobulin lambda-like polypeptide 1 | IGL@ /// IGLL1 | 505478 /// 789205 | 0.7017 | 0.0161 |
| Bt_9599_1_A1_at | Bt.49510 | Inositol hexaphosphate kinase 2 | IHPK2 | 508236 | 0.6362 | 0.0353 |
| Bt_24532_2_S1_at | Bt.24532 | interleukin 17 receptor B | IL17RB | 533605 | -0.8924 | 0.0002 |
| Bt_22316_1_S1_at | Bt.26801 | IQ motif containing C | IQCC | 507606 | -0.7601 | 0.0066 |
| Bt_19782_2_S1_a_at | Bt.19782 | interleukin-1 receptor-associated kinase 1 binding protein 1 | IRAK1BP1 | 782235 | -0.7086 | 0.0147 |
| Bt_15806_2_A1_at | --- | iron-responsive element binding protein 2 | IREB2 | 539404 | -0.6236 | 0.0404 |
| Bt_15989_1_A1_at | Bt.15989 | interferon regulatory factor 2 | IRF2 | 337916 | -0.6515 | 0.0299 |
| Bt_20182_1_S1_at | Bt.45332 | interferon regulatory factor 3 | IRF3 | 516979 | 0.6753 | 0.0226 |
| Bt_22275_1_A1_at | Bt.22275 | interferon stimulated exonuclease gene 20kDa | ISG20 | 506604 | 0.6387 | 0.0344 |
| Bt_29920_1_S1_at | Bt.80589 | interferon stimulated exonuclease gene 20kDa-like 2 | ISG20L2 | 515980 | -0.6983 | 0.0168 |
| Bt_27978_1_A1_at | Bt.35830 | integrin, alpha M (complement component 3 receptor 3 subunit) | ITGAM | 407124 | -0.7660 | 0.0060 |
| Bt_21658_1_S1_at | Bt.21658 | inositol 1,4,5-trisphosphate 3-kinase C | ITPKC | 534051 | 0.6424 | 0.0330 |
| Bt_22721_1_A1_a_at | Bt.59662 | JNK1/MAPK8-associated membrane protein | JKAMP | 509181 | 0.6282 | 0.0385 |
| Bt_6605_1_S1_x_at | Bt.1255 | jumping translocation breakpoint | JTB | 513970 | 0.6809 | 0.0211 |
| Bt_4780_1_S1_at | Bt.77226 | K(lysine) acetyltransferase 5 | KAT5 | 505619 | 0.7135 | 0.0137 |
| Bt_13065_1_S1_at | Bt.31577 | potassium inwardly-rectifying channel, subfamily J, member 8 | KCNJ8 | 282572 | 0.6806 | 0.0212 |
| Bt_13860_2_A1_at | Bt.13860 | kinase insert domain receptor (a type III receptor tyrosine kinase) | KDR | 407170 | -0.7022 | 0.0160 |
| Bt_10543_1_S1_at | Bt.26095 | Hypothetical protein LOC784675 | KIAA1462 | 784675 | -0.6057 | 0.0483 |
| Bt_24248_1_S1_at | Bt.53718 | KIAA1737 | KIAA1737 | 506130 | -0.7087 | 0.0146 |
| Bt_29866_1_S1_at | Bt.89096 | killer cell immunoglobulin-like receptor, three domains, short cytoplasmic tail, 1 | KIR3DS1 | 493736 | -0.7329 | 0.0103 |
| Bt_5626_2_S1_at | Bt.48334 | Kinesin light chain 1 | KLC1 | 508999 | 0.6389 | 0.0343 |
| Bt_25044_1_S1_at | --- | karyopherin alpha 4 (importin alpha 3) | KPNA4 | 535090 | 0.6600 | 0.0271 |
| Bt_13421_3_S1_at | Bt.13421 | KRI1 homolog (S. cerevisiae) | KRI1 | 511427 | -0.6343 | 0.0361 |
| Bt_23465_1_S1_at | Bt.35657 | keratin 25 | KRT25 | 511540 | -0.6241 | 0.0401 |
| Bt_3719_1_S1_at | Bt.3719 | L antigen family, member 3 | LAGE3 | 782436 | -0.6494 | 0.0306 |
| Bt_27230_1_S1_at | Bt.54919 | laminin, gamma 3 | LAMC3 | 518914 | 0.7578 | 0.0069 |
| Bt_15878_1_S1_at | Bt.63555 | LanC lantibiotic synthetase component C-like 1 (bacterial) | LANCL1 | 540559 | -0.6446 | 0.0323 |
| Bt_27891_1_S1_at | Bt.59370 | leucyl-tRNA synthetase 2, mitochondrial | LARS2 | 504609 | -0.6478 | 0.0311 |
| Bt_2268_1_S1_at | Bt.2268 | LAG1 homolog, ceramide synthase 4 | LASS4 | 505233 | -0.6434 | 0.0327 |
| Bt_17942_1_S1_at | Bt.17942 | limb bud and heart development homolog (mouse) | LBH | 616148 | -0.6269 | 0.0390 |
| Bt_6059_1_S1_at | Bt.42836 | leucine carboxyl methyltransferase 1 | LCMT1 | 618021 | -0.6135 | 0.0447 |
| Bt_27048_1_A1_at | Bt.27048 | Lactase-like | LCTL | 518599 | -0.7379 | 0.0095 |
| Bt_4553_2_S1_at | Bt.4553 | LIM domain binding 1 | LDB1 | 526472 | -0.6441 | 0.0324 |
| Bt_195_1_A1_at | Bt.33536 | leukocyte cell-derived chemotaxin 2 | LECT2 | 281899 | -0.6144 | 0.0443 |
| Bt_13415_1_A1_at | Bt.38540 | leptin receptor overlapping transcript-like 1 | LEPROTL1 | 513036 | 0.7344 | 0.0101 |
| Bt_24718_1_S1_at | Bt.24718 | LETM1 domain containing 1 | LETMD1 | 514595 | -0.6246 | 0.0399 |
| Bt_26621_1_S1_at | Bt.35117 | leucine-rich, glioma inactivated 1 | LGI1 | 617080 | -0.6673 | 0.0249 |
| Bt_9756_1_S1_at | Bt.9756 | lipoic acid synthetase | LIAS | 530865 | -0.6034 | 0.0494 |
| Bt_26499_1_A1_at | Bt.43873 | lin-52 homolog (C. elegans) | LIN52 | 618104 | -0.6335 | 0.0364 |
| Bt_25014_1_A1_at | Bt.25014 | lin-7 homolog B (C. elegans) | LIN7B | 616283 | -0.6935 | 0.0179 |
| Bt_25951_1_A1_at | Bt.52154 | Lethal giant larvae homolog 2 (Drosophila) | LLGL2 | 539545 | -0.6590 | 0.0274 |
| Bt_4643_1_S1_at | Bt.76431 | lectin, mannose-binding 2 | LMAN2 | 790870 | 0.6098 | 0.0464 |
| Bt_19850_2_S1_at | Bt.89521 | Similar to Long-chain-fatty-acid--CoA ligase 3 (Long-chain acyl-CoA synthetase 3) (LACS 3) | LOC100138312 | 100138312 | -0.7175 | 0.0129 |
| Bt_18204_2_A1_at | --- | hypothetical protein LOC100139162 | LOC100139162 | 100139162 | -0.6849 | 0.0200 |
| Bt_16755_1_A1_at | --- | similar to mCG67939 | LOC100139345 | 100139345 | -0.7228 | 0.0120 |
| Bt_20947_1_S1_at | Bt.2500 | Similar to IQ motif containing GTPase activating protein 2 | LOC100140708 | 100140708 | -0.7008 | 0.0163 |
| Bt_9565_1_S1_at | Bt.9565 | histone H2B variant PT15 | LOC404073 | 404073 | -0.6706 | 0.0239 |
| Bt_28518_1_S1_s_at | Bt.102246 | spleen trypsin inhibitor /// pancreatic trypsin inhibitor | LOC404103 /// PTI | 404103 /// 404172 | -0.7111 | 0.0141 |
| Bt_29682_1_A1_at | Bt.92690 | T cell receptor delta chain | LOC407199 | 407199 | -0.6719 | 0.0236 |
| Bt_27999_1_S1_at | Bt.96926 | IgM | LOC444875 | 444875 | 0.6988 | 0.0167 |
| Bt_6372_1_A1_at | Bt.37782 | similar to cationic amino acid transporter 5 | LOC504861 | 504861 | 0.6656 | 0.0254 |
| Bt_4846_1_S1_at | Bt.4846 | similar to CG8043 CG8043-PA | LOC506009 | 506009 | -0.7700 | 0.0056 |
| Bt_3444_1_A1_at | Bt.91052 | Similar to TATA-binding protein-like factor-interacting protein | LOC506099 | 506099 | -0.6757 | 0.0225 |
| Bt_2434_1_A1_at | Bt.2434 | similar to family with sequence similarity 132, member A | LOC506102 | 506102 | -0.7635 | 0.0062 |
| Bt_13314_1_S1_at | --- | similar to Transcription factor COE4 (Early B-cell factor 4) (EBF-4) (Olf-1/EBF-like 4) (OE-4) (O/E-4) | LOC506470 | 506470 | -0.6403 | 0.0338 |
| Bt_24799_1_S1_at | --- | similar to poly (ADP-ribose) polymerase family, member 3 | LOC507426 | 507426 | 0.6306 | 0.0375 |
| Bt_25872_1_A1_at | Bt.52183 | Similar to G protein-coupled receptor 98 | LOC507513 | 507513 | -0.6084 | 0.0470 |
| Bt_25863_1_A1_at | Bt.25863 | Similar to olfactory receptor 1510 | LOC507605 | 507605 | -0.7202 | 0.0124 |
| Bt_26538_1_S1_at | Bt.26538 | similar to chromosome 9 open reading frame 61 | LOC509420 | 509420 | -0.8144 | 0.0023 |
| Bt_1800_1_S1_at | Bt.38598 | Hypothetical LOC510385 | LOC510385 | 510385 | -0.6993 | 0.0166 |
| Bt_10716_2_S1_at | Bt.10716 | Hypothetical protein LOC510660 | LOC510660 | 510660 | -0.6470 | 0.0314 |
| Bt_27392_1_A1_at | Bt.66112 | hypothetical LOC510961 | LOC510961 | 510961 | -0.6036 | 0.0493 |
| Bt_24983_1_A1_at | Bt.53810 | similar to dynein, cytoplasmic 2, heavy chain 1 | LOC512287 | 512287 | -0.7699 | 0.0056 |
| Bt_17158_2_S1_at | Bt.17158 | similar to Uncharacterized protein KIAA0753 | LOC512933 | 512933 | -0.7043 | 0.0155 |
| Bt_2033_1_A1_at | Bt.10124 | Hypothetical LOC513129 | LOC513129 | 513129 | -0.6989 | 0.0167 |
| Bt_26750_1_S1_at | Bt.65066 | similar to RIKEN cDNA 4832428D23 | LOC513822 | 513822 | -0.6221 | 0.0410 |
| Bt_27001_1_S1_at | Bt.9071 | Hypothetical protein LOC514154 | LOC514154 | 514154 | 0.6739 | 0.0230 |
| Bt_26121_1_S1_at | --- | similar to FERM, RhoGEF and pleckstrin domain protein 2 | LOC514637 | 514637 | -0.6515 | 0.0299 |
| Bt_20051_1_S1_at | --- | similar to SOCIUS protein | LOC515112 | 515112 | -0.6536 | 0.0292 |
| Bt_19355_1_A1_at | Bt.19355 | Similar to nucleoporin 62 | LOC516074 | 516074 | -0.6039 | 0.0491 |
| Bt_7030_1_S1_at | --- | similar to SIN3 homolog B, transcription regulator (yeast) | LOC520505 | 520505 | -0.6112 | 0.0457 |
| Bt_28626_2_S1_at | --- | similar to GC-rich sequence DNA-binding factor (GCF) (Transcription factor 9) (TCF-9) | LOC521363 | 521363 | 0.6760 | 0.0224 |
| Bt_23486_2_S1_at | Bt.23486 | similar to myosin IXA | LOC521389 | 521389 | -0.7647 | 0.0061 |
| Bt_25911_1_A1_at | Bt.87197 | Similar to hCG1788238 | LOC521943 | 521943 | -0.6443 | 0.0324 |
| Bt_26734_1_A1_at | --- | similar to Semaphorin-6C precursor (Semaphorin-Y) (Sema Y) | LOC524464 | 524464 | -0.6322 | 0.0369 |
| Bt_1848_2_A1_at | --- | similar to tubulin tyrosine ligase-like family, member 6 | LOC526482 | 526482 | -0.6322 | 0.0369 |
| Bt_24642_1_A1_at | Bt.87955 | similar to ankyrin repeat domain 26 /// similar to ankyrin repeat domain 26 | LOC526505 /// LOC785905 | 526505 /// 785905 | -0.6542 | 0.0290 |
| Bt_18630_1_A1_at | Bt.31790 | similar to hCG1812738 | LOC528523 | 528523 | -0.7069 | 0.0150 |
| Bt_9722_1_S1_at | Bt.57437 | similar to CG3558 CG3558-PA | LOC528939 | 528939 | 0.7803 | 0.0046 |
| Bt_13254_1_S1_at | --- | similar to family with sequence similarity 40, member B | LOC529423 | 529423 | -0.7777 | 0.0048 |
| Bt_5769_1_S1_at | Bt.48596 | Similar to AAT1-alpha | LOC531600 | 531600 | -0.6814 | 0.0209 |
| Bt_27140_1_S1_at | --- | similar to mKIAA1077 protein | LOC535166 | 535166 | 0.6223 | 0.0409 |
| Bt_29208_1_S1_at | Bt.29208 | similar to KIAA0715 protein | LOC535967 | 535967 | 0.6471 | 0.0314 |
| Bt_20273_2_S1_a_at | --- | similar to patatin-like phospholipase domain containing 7 | LOC536255 | 536255 | -0.6351 | 0.0358 |
| Bt_10842_2_S1_a_at | Bt.42175 | similar to cytidine monophosphate-N-acetylneuraminic acid hydroxylase | LOC537017 | 537017 | 0.6299 | 0.0378 |
| Bt_27622_1_A1_at | Bt.27622 | Similar to myosin tail domain-containing protein | LOC538331 | 538331 | -0.6705 | 0.0240 |
| Bt_16624_1_A1_at | Bt.16624 | Similar to Protein FAM81A | LOC538402 | 538402 | -0.6589 | 0.0274 |
| Bt_21019_1_S1_at | --- | similar to Protein capicua homolog | LOC538483 | 538483 | -0.6585 | 0.0276 |
| Bt_21937_1_A1_at | --- | similar to hepatitis C virus F protein-binding protein 2 | LOC539495 | 539495 | -0.7752 | 0.0051 |
| Bt_3990_1_S1_at | Bt.3990 | hypothetical protein LOC540222 | LOC540222 | 540222 | -0.6797 | 0.0214 |
| Bt_16776_1_S1_at | Bt.60437 | similar to tigger transposable element derived 5 | LOC540422 | 540422 | -0.7033 | 0.0157 |
| Bt_1978_14_A1_at | Bt.61335 | T-cell receptor beta chain V region | LOC613389 | 613389 | -0.8021 | 0.0030 |
| Bt_10169_2_S1_at | Bt.10169 | similar to Uncharacterized protein C8orf42 homolog | LOC614166 | 614166 | -0.6335 | 0.0364 |
| Bt_12646_1_A1_at | Bt.12646 | Similar to histone cluster 1, H2bd | LOC614376 | 614376 | -0.6038 | 0.0492 |
| Bt_1907_1_S1_at | Bt.1907 | hypothetical LOC614490 | LOC614490 | 614490 | -0.6551 | 0.0287 |
| Bt_18932_1_A1_at | Bt.47932 | hypothetical protein LOC614993 | LOC614993 | 614993 | -0.6422 | 0.0331 |
| Bt_28707_1_S1_at | --- | similar to BAI1-associated protein 2-like 1 | LOC615412 | 615412 | -0.7658 | 0.0060 |
| Bt_26632_1_S1_at | Bt.26632 | similar to chromosome 10 open reading frame 76 | LOC616557 | 616557 | -0.6493 | 0.0306 |
| Bt_27222_1_A1_at | Bt.27222 | Similar to Uncharacterized protein C20orf26 | LOC616648 | 616648 | -0.6527 | 0.0295 |
| Bt_9349_1_S1_at | --- | similar to coagulation factor VIII-associated protein | LOC617475 | 617475 | -0.6221 | 0.0410 |
| Bt_10616_1_S1_a_at | --- | similar to chromosome 6 open reading frame 52 | LOC618944 | 618944 | -0.7250 | 0.0116 |
| Bt_454_1_S1_at | Bt.454 | prolactin-related protein 12 /// prolactin-related protein 1 | LOC751563 /// PRP1 | 281429 /// 751563 | -0.6569 | 0.0281 |
| Bt_18264_1_A1_at | Bt.18264 | similar to RIKEN cDNA 2200001I15 | LOC781772 | 781772 | -0.6163 | 0.0435 |
| Bt_27589_1_A1_at | --- | similar to hCG1779312 | LOC781795 | 781795 | -0.8259 | 0.0017 |
| Bt_9224_1_S1_at | Bt.9224 | Similar to LOC152217 protein | LOC783161 | 783161 | -0.6776 | 0.0220 |
| Bt_13796_1_S1_at | Bt.64369 | similar to Equ c1 | LOC783399 | 783399 | -0.7323 | 0.0104 |
| Bt_24780_1_S1_at | --- | similar to AT rich interactive domain 5B (MRF1-like) | LOC783538 | 783538 | 0.6666 | 0.0251 |
| Bt_29496_1_A1_at | Bt.102171 | similar to Uncharacterized protein C1orf112 homolog | LOC784034 | 784034 | -0.7038 | 0.0156 |
| Bt_12818_1_S1_at | Bt.12818 | similar to Testis-specific Y-encoded protein 1 (bTSPY) /// similar to Testis-specific Y-encoded protein 1 (bTSPY) /// similar to Testis-specific Y-encoded protein 1 (bTSPY) /// similar to Testis-specific Y-encoded protein 1 (bTSPY) /// testis-specific pro | LOC784849 /// LOC785348 /// LOC785571 /// LOC785608 /// TSPY | 281554 /// 784849 /// 785348 /// 785571 /// 785608 | -0.7371 | 0.0096 |
| Bt_6362_1_A1_at | --- | similar to lipase, hormone-sensitive | LOC785088 | 785088 | -0.7393 | 0.0093 |
| Bt_13003_5_S1_at | Bt.89591 | hypothetical protein LOC785621 | LOC785621 | 785621 | -0.7155 | 0.0133 |
| Bt_27486_1_A1_at | Bt.27486 | Similar to Uncharacterized protein C20orf26 | LOC786312 | 786312 | -0.7142 | 0.0135 |
| Bt_17329_1_A1_at | Bt.65119 | similar to hCG1777421 | LOC787439 | 787439 | -0.6936 | 0.0179 |
| Bt_1443_1_A1_at | Bt.65108 | similar to Uncharacterized protein C22orf30 | LOC787908 | 787908 | -0.7424 | 0.0089 |
| Bt_20232_3_S1_at | --- | similar to Snf2-related CBP activator protein | LOC788113 | 788113 | 0.6280 | 0.0385 |
| Bt_17036_1_A1_s_at | Bt.43956 | similar to WARS protein /// tryptophanyl-tRNA synthetase | LOC790042 /// WARS | 281576 /// 790042 | 0.6241 | 0.0401 |
| Bt_7555_2_S1_at | Bt.7555 | similar to stromal membrane-associated GTPase-activating protein 2 /// small ArfGAP2 | LOC790140 /// SMAP2 | 514465 /// 790140 | 0.6311 | 0.0373 |
| Bt_27869_1_A1_at | Bt.65320 | lysophosphatidic acid receptor 2 | LPAR2 | 509748 | -0.6640 | 0.0259 |
| Bt_26573_2_S1_at | Bt.26573 | lysophosphatidylcholine acyltransferase 3 | LPCAT3 | 515361 | -0.6902 | 0.0187 |
| Bt_24498_1_S1_at | Bt.55363 | leucine rich repeat and fibronectin type III domain containing 3 | LRFN3 | 767839 | 0.6293 | 0.0380 |
| Bt_22664_1_A1_at | Bt.30792 | leucine-rich repeats and immunoglobulin-like domains 3 | LRIG3 | 506574 | -0.7766 | 0.0049 |
| Bt_29862_1_A1_at | Bt.102062 | low density lipoprotein receptor-related protein 8, apolipoprotein e receptor | LRP8 | 407179 | -0.6337 | 0.0363 |
| Bt_26880_1_A1_at | --- | leucine rich repeat containing 45 | LRRC45 | 514524 | -0.6522 | 0.0296 |
| Bt_26160_1_A1_at | Bt.25557 | maelstrom homolog (Drosophila) | MAEL | 541073 | -0.8844 | 0.0003 |
| Bt_13587_1_S1_at | Bt.13587 | Mannosidase, alpha, class 2A, member 1 | MAN2A1 | 521539 | 0.7487 | 0.0080 |
| Bt_3516_1_S1_at | Bt.56314 | mannosidase, alpha, class 2A, member 2 | MAN2A2 | 527449 | 0.6671 | 0.0249 |
| Bt_23082_1_S1_at | Bt.23082 | mitogen-activated protein kinase kinase kinase kinase 2 | MAP4K2 | 520058 | -0.6196 | 0.0420 |
| Bt_22442_3_S1_a_at | Bt.52956 | mitogen-activated protein kinase 12 | MAPK12 | 512943 | -0.6316 | 0.0371 |
| Bt_3448_2_S1_at | Bt.63187 | Mitogen-activated protein kinase 6 | MAPK6 | 538094 | 0.6832 | 0.0205 |
| Bt_26589_1_S1_at | Bt.4121 | membrane-associated ring finger (C3HC4) 6 | Mar-06 | 540371 | 0.6127 | 0.0451 |
| Bt_8182_2_S1_at | Bt.8182 | membrane-associated ring finger (C3HC4) 8 | Mar-08 | 540667 | 0.6094 | 0.0465 |
| Bt_18862_2_S1_at | Bt.5646 | membrane-associated ring finger (C3HC4) 9 | Mar-09 | 540871 | -0.6999 | 0.0165 |
| Bt_25149_1_A1_at | Bt.35261 | Matrin 3 | MATR3 | 505129 | -0.6770 | 0.0221 |
| Bt_8671_1_S1_at | Bt.8671 | metallo-beta-lactamase domain containing 2 | MBLAC2 | 537692 | -0.6082 | 0.0471 |
| Bt_6405_1_S1_at | Bt.64741 | myelin basic protein | MBP | 618684 | 0.6906 | 0.0186 |
| Bt_27954_1_A1_at | Bt.77376 | mbt domain containing 1 | MBTD1 | 511415 | -0.6290 | 0.0382 |
| Bt_3956_1_S2_at | Bt.3956 | melanocortin 2 receptor (adrenocorticotropic hormone) | MC2R | 281299 | -0.7050 | 0.0154 |
| Bt_12661_1_A1_at | Bt.12661 | methylcrotonoyl-Coenzyme A carboxylase 1 (alpha) | MCCC1 | 513504 | -0.6829 | 0.0206 |
| Bt_951_1_S1_at | Bt.59074 | myeloid cell leukemia sequence 1 (BCL2-related) | MCL1 | 788087 | 0.6872 | 0.0195 |
| Bt_25082_1_A1_at | Bt.45508 | Mucolipin 3 | MCOLN3 | 514345 | -0.7072 | 0.0149 |
| Bt_8938_1_S1_at | Bt.8938 | MD-2 protein | MD-2 | 281304 | -0.6073 | 0.0475 |
| Bt_27218_1_A1_at | Bt.27218 | methyl CpG binding protein 2 (Rett syndrome) | MECP2 | 539629 | 0.7580 | 0.0069 |
| Bt_1348_1_S1_at | Bt.1348 | mediator complex subunit 17 | MED17 | 541303 | -0.6462 | 0.0317 |
| Bt_5685_1_S1_at | Bt.5685 | mediator complex subunit 22 | MED22 | 613736 | -0.7361 | 0.0098 |
| Bt_9874_1_S1_at | Bt.12647 | Mediator complex subunit 25 | MED25 | 533865 | -0.7436 | 0.0087 |
| Bt_28755_1_S1_at | Bt.61520 | mediator complex subunit 26 | MED26 | 506331 | -0.7608 | 0.0065 |
| Bt_22622_2_S1_at | Bt.22622 | mediator complex subunit 30 | MED30 | 613879 | -0.6386 | 0.0344 |
| Bt_24579_1_S1_at | Bt.88324 | mediator complex subunit 31 | MED31 | 532868 | -0.6619 | 0.0265 |
| Bt_16150_1_A1_at | Bt.6869 | Myocyte enhancer factor 2C | MEF2C | 512254 | 0.6654 | 0.0254 |
| Bt_25390_1_A1_at | Bt.43191 | Meis homeobox 1 | MEIS1 | 613877 | -0.7396 | 0.0093 |
| Bt_9633_2_S1_at | Bt.63482 | mesenchyme homeobox 2 | MEOX2 | 518540 | -0.6372 | 0.0350 |
| Bt_29873_1_A1_at | Bt.22540 | mesoderm specific transcript homolog (mouse) | MEST | 404180 | -0.6526 | 0.0295 |
| Bt_28565_1_S1_at | Bt.28565 | methyltransferase 5 domain containing 1 | METT5D1 | 533987 | -0.6315 | 0.0372 |
| Bt_27078_1_A1_at | Bt.13303 | Methyltransferase like 3 | METTL3 | 540339 | 0.6427 | 0.0329 |
| Bt_25441_1_A1_at | Bt.52288 | Microfibrillar-associated protein 1 | MFAP1 | 510905 | -0.7396 | 0.0093 |
| Bt_3092_1_S1_at | Bt.22115 | microfibrillar-associated protein 2 | MFAP2 | 281912 | 0.6064 | 0.0479 |
| Bt_27274_1_A1_at | Bt.27274 | major facilitator superfamily domain containing 2 | MFSD2 | 512633 | -0.6246 | 0.0400 |
| Bt_19043_2_A1_at | Bt.64720 | Mannosyl (alpha-1,6-)-glycoprotein beta-1,6-N-acetyl-glucosaminyltransferase, isozyme B | MGAT5B | 785213 | -0.6878 | 0.0193 |
| Bt_24155_1_A1_at | Bt.52665 | hypothetical LOC508280 | MGC127461 | 508280 | -0.7248 | 0.0116 |
| Bt_22468_1_S1_at | Bt.89095 | hypothetical LOC510399 | MGC128008 | 510399 | -0.7586 | 0.0068 |
| Bt_11245_1_S1_at | Bt.49479 | hypothetical protein MGC128424 | MGC128424 | 767924 | 0.6749 | 0.0227 |
| Bt_17723_1_A1_at | Bt.68057 | Hypothetical protein MGC133619 | MGC133619 | 767960 | -0.6173 | 0.0430 |
| Bt_17517_1_S1_at | Bt.61307 | Hypothetical LOC505226 | MGC134574 | 505226 | -0.6180 | 0.0427 |
| Bt_9213_1_A1_at | Bt.9213 | Fanconi anemia-associated protein, 24 kDa | MGC137368 | 510793 | -0.6474 | 0.0313 |
| Bt_6962_1_S1_at | Bt.6962 | hypothetical protein LOC613997 | MGC139355 | 613997 | -0.6966 | 0.0172 |
| Bt_28608_1_S1_at | Bt.28608 | Similar to calpain | MGC151839 | 617138 | -0.6422 | 0.0331 |
| Bt_13422_2_A1_at | Bt.13422 | Hypothetical LOC507035 | MGC152585 | 507035 | -0.6176 | 0.0429 |
| Bt_9793_1_A1_at | Bt.9793 | Hypothetical LOC511431 | MGC160092 | 511431 | -0.8278 | 0.0017 |
| Bt_20600_2_A1_a_at | Bt.59196 | MYC induced nuclear antigen | MINA | 540466 | -0.6691 | 0.0243 |
| Bt_3678_1_S1_at | Bt.53167 | MKI67 (FHA domain) interacting nucleolar phosphoprotein | MKI67IP | 509598 | -0.6093 | 0.0466 |
| Bt_13707_1_A1_at | Bt.20614 | myeloid/lymphoid or mixed-lineage leukemia 2 | MLL2 | 506805 | -0.6422 | 0.0331 |
| Bt_28550_1_S1_at | --- | similar to Myeloid/lymphoid or mixed-lineage leukemia protein 3 homolog (Histone-lysine N-methyltransferase, H3 lysine-4 specific MLL3) (Homologous to ALR protein) (Lysine N-methyltransferase 2C) | MLL3 | 789336 | -0.7089 | 0.0146 |
| Bt_13347_1_S1_at | Bt.46245 | myeloid/lymphoid or mixed-lineage leukemia (trithorax homolog, Drosophila); translocated to, 1 | MLLT1 | 504458 | -0.6322 | 0.0369 |
| Bt_24873_1_S1_at | Bt.38559 | Myeloid/lymphoid or mixed-lineage leukemia (trithorax homolog, Drosophila); translocated to, 10 | MLLT10 | 519864 | -0.6275 | 0.0388 |
| Bt_18585_1_S1_at | Bt.45494 | Myeloid/lymphoid or mixed-lineage leukemia (trithorax homolog, Drosophila); translocated to, 4 | MLLT4 | 504856 | -0.7652 | 0.0061 |
| Bt_18079_1_A1_at | Bt.46080 | Malonyl-CoA decarboxylase | MLYCD | 512341 | -0.6588 | 0.0275 |
| Bt_18504_1_S1_at | Bt.18504 | matrix metallopeptidase 3 (stromelysin 1, progelatinase) | MMP3 | 281309 | 0.7257 | 0.0115 |
| Bt_3900_1_S1_at | Bt.15978 | molybdenum cofactor synthesis 1 | MOCS1 | 281917 | 0.6315 | 0.0372 |
| Bt_18526_1_A1_at | Bt.48324 | MORN repeat containing 1 | MORN1 | 617176 | -0.7267 | 0.0113 |
| Bt_3319_1_A1_at | Bt.3319 | MORN repeat containing 4 | MORN4 | 614519 | -0.6765 | 0.0223 |
| Bt_25277_1_A1_at | Bt.55002 | multiple PDZ domain protein | MPDZ | 536863 | -0.6683 | 0.0246 |
| Bt_27187_1_S1_at | Bt.52586 | M-phase phosphoprotein 10 (U3 small nucleolar ribonucleoprotein) | MPHOSPH10 | 531149 | -0.7024 | 0.0159 |
| Bt_23901_1_A1_at | Bt.23901 | Myeloperoxidase | MPO | 511206 | -0.6117 | 0.0455 |
| Bt_20102_1_S1_at | Bt.20102 | MPV17 mitochondrial membrane protein-like 2 | MPV17L2 | 618791 | 0.6330 | 0.0366 |
| Bt_3829_2_S1_at | Bt.63045 | major histocompatibility complex, class I-related | MR1 | 506206 | 0.7279 | 0.0111 |
| Bt_10874_1_S1_at | Bt.10874 | methylthioribose-1-phosphate isomerase homolog (S. cerevisiae) | MRI1 | 534734 | -0.6087 | 0.0469 |
| Bt_15953_1_S1_at | Bt.15953 | mitochondrial ribosomal protein L14 | MRPL14 | 614579 | -0.6510 | 0.0301 |
| Bt_13908_1_S1_at | Bt.16083 | mitochondrial ribosomal protein S22 | MRPS22 | 532044 | -0.7529 | 0.0075 |
| Bt_1674_2_S1_at | Bt.49027 | mitochondrial ribosomal protein S34 | MRPS34 | 618357 | -0.7337 | 0.0102 |
| Bt_24624_1_S1_at | Bt.53127 | mitochondrial ribosomal protein S9 | MRPS9 | 519302 | -0.8823 | 0.0003 |
| Bt_9207_1_S1_at | Bt.9207 | moesin | MSN | 540426 | 0.6336 | 0.0363 |
| Bt_23042_1_S1_at | Bt.102450 | metallothionein 1E | MT1E | 404071 | 0.6212 | 0.0413 |
| Bt_2529_1_S1_at | Bt.2529 | metallothionein 2A | MT2A | 404070 | 0.6143 | 0.0443 |
| Bt_22983_1_S1_at | Bt.22983 | metal-regulatory transcription factor 1 | MTF1 | 509960 | 0.6022 | 0.0499 |
| Bt_17746_1_A1_at | Bt.28705 | Myotubularin related protein 2 | MTMR2 | 536810 | -0.7648 | 0.0061 |
| Bt_15819_2_S1_at | Bt.15819 | metaxin 2 | MTX2 | 615424 | -0.6462 | 0.0317 |
| Bt_11033_1_S1_at | Bt.43056 | Melanoma associated antigen (mutated) 1 | MUM1 | 513471 | -0.6528 | 0.0294 |
| Bt_29010_1_A1_at | Bt.48853 | Major vault protein | MVP | 516456 | -0.6120 | 0.0454 |
| Bt_23229_2_S1_at | Bt.25860 | myosin binding protein C, slow type | MYBPC1 | 510763 | -0.6515 | 0.0299 |
| Bt_29854_1_S1_x_at | Bt.12300 | Myosin, heavy chain 1, skeletal muscle, adult | MYH1 | 281337 | -0.7845 | 0.0043 |
| Bt_23497_1_S1_at | Bt.23497 | myosin, light chain 3, alkali; ventricular, skeletal, slow | MYL3 | 618352 | -0.6566 | 0.0282 |
| Bt_28807_1_S1_at | Bt.80327 | N-acetylglutamate synthase | NAGS | 538004 | -0.6909 | 0.0186 |
| Bt_16099_2_S1_at | --- | NMDA receptor regulated 1 | NARG1 | 536676 | -0.6210 | 0.0414 |
| Bt_15937_1_A1_at | Bt.52304 | N-acetyltransferase 11 (GCN5-related, putative) | NAT11 | 517933 | -0.6841 | 0.0203 |
| Bt_21092_1_S1_at | Bt.58167 | nudE nuclear distribution gene E homolog (A. nidulans)-like 1 | NDEL1 | 510769 | 0.6842 | 0.0202 |
| Bt_17970_2_A1_at | Bt.11553 | NIMA (never in mitosis gene a)-related kinase 3 | NEK3 | 505876 | 0.6255 | 0.0396 |
| Bt_21763_3_S1_at | Bt.91925 | nuclear factor I/B | NFIB | 538474 | -0.6088 | 0.0468 |
| Bt_27854_2_S1_at | Bt.27854 | nuclear factor, interleukin 3 regulated | NFIL3 | 506097 | 0.6978 | 0.0170 |
| Bt_23207_1_A1_s_at | Bt.8227 | nuclear factor of kappa light polypeptide gene enhancer in B-cells inhibitor, zeta | NFKBIZ | 282713 | 0.6546 | 0.0288 |
| Bt_3023_1_S1_at | Bt.96669 | nitrilase 1 | NIT1 | 504199 | -0.6917 | 0.0184 |
| Bt_27987_1_S1_at | Bt.27987 | tachikinin receptor 1 | nk1R | 407133 | -0.6234 | 0.0405 |
| Bt_10277_1_A1_at | Bt.49005 | NOP56 ribonucleoprotein homolog (yeast) | NOP56 | 404165 | 0.6625 | 0.0263 |
| Bt_27958_1_A1_at | --- | nephronophthisis 4 | NPHP4 | 519551 | -0.6656 | 0.0254 |
| Bt_3367_1_S1_at | Bt.44371 | nuclear receptor subfamily 1, group H, member 2 | NR1H2 | 509622 | 0.6714 | 0.0237 |
| Bt_22888_1_S1_at | Bt.22888 | nuclear receptor subfamily 3, group C, member 1 (glucocorticoid receptor) | NR3C1 | 281946 | -0.6418 | 0.0333 |
| Bt_29769_1_S1_x_at | Bt.87895 | nuclear receptor subfamily 5, group A, member 1 | NR5A1 | 281948 | -0.6091 | 0.0467 |
| Bt_22053_1_S1_at | Bt.22053 | nuclear receptor binding protein 2 | NRBP2 | 504664 | -0.6036 | 0.0493 |
| Bt_411_1_S1_at | Bt.411 | neuregulin 1 | NRG1 | 281361 | 0.7207 | 0.0123 |
| Bt_727_3_S1_a_at | Bt.64628 | nucleoporin 37kDa | NUP37 | 100139879 | -0.6343 | 0.0361 |
| Bt_28119_1_S1_at | Bt.15537 | Outer dense fiber of sperm tails 2 | ODF2 | 539043 | -0.6465 | 0.0316 |
| Bt_114_1_S1_at | Bt.114 | osteomodulin | OMD | 280885 | -0.6937 | 0.0179 |
| Bt_242_1_S1_at | Bt.242 | opioid receptor, mu 1 | OPRM1 | 281958 | -0.6973 | 0.0171 |
| Bt_24267_1_A1_at | Bt.24267 | origin recognition complex, subunit 3-like (yeast) | ORC3L | 523714 | -0.6389 | 0.0343 |
| Bt_13189_2_S1_at | Bt.13189 | origin recognition complex, subunit 4-like (yeast) | ORC4L | 529245 | -0.6403 | 0.0338 |
| Bt_23801_1_A1_at | Bt.12578 | Origin recognition complex, subunit 5-like (yeast) | ORC5L | 519409 | -0.6738 | 0.0230 |
| Bt_27676_1_S1_at | Bt.27676 | OTU domain, ubiquitin aldehyde binding 2 | OTUB2 | 504880 | 0.6200 | 0.0419 |
| Bt_21584_1_S1_at | Bt.21584 | 3-oxoacyl-ACP synthase, mitochondrial | OXSM | 513530 | -0.6848 | 0.0201 |
| Bt_16016_1_S1_at | Bt.34356 | purinergic receptor P2X, ligand-gated ion channel, 5 | P2RX5 | 338035 | -0.7105 | 0.0143 |
| Bt_13024_1_A1_at | Bt.13024 | purinergic receptor P2Y, G-protein coupled, 2 | P2RY2 | 282638 | -0.6862 | 0.0197 |
| Bt_20356_1_S1_at | Bt.20356 | Proapoptotic caspase adapter protein | PACAP | 510480 | 0.6532 | 0.0293 |
| Bt_25419_1_A1_at | Bt.61691 | PARK2 co-regulated | PACRG | 767959 | -0.6673 | 0.0249 |
| Bt_288_1_S1_at | Bt.288 | pregnancy-associated glycoprotein 15 | PAG15 | 337909 | -0.8628 | 0.0006 |
| Bt_22106_1_A1_at | Bt.17706 | P21 protein (Cdc42/Rac)-activated kinase 1 | PAK1 | 533729 | -0.6760 | 0.0224 |
| Bt_18229_1_A1_at | Bt.51708 | partner and localizer of BRCA2 | PALB2 | 507620 | -0.7048 | 0.0154 |
| Bt_4936_1_S1_at | Bt.4936 | paralemmin | PALM | 786096 | -0.6184 | 0.0425 |
| Bt_23827_1_A1_at | Bt.59483 | Poly(A) polymerase alpha | PAPOLA | 338051 | -0.6121 | 0.0453 |
| Bt_2763_1_S1_at | Bt.65122 | poly (ADP-ribose) glycohydrolase | PARG | 281377 | -0.7120 | 0.0140 |
| Bt_24585_3_A1_at | Bt.24585 | Poly (ADP-ribose) polymerase family, member 10 | PARP10 | 510991 | 0.7426 | 0.0088 |
| Bt_26533_1_S1_at | Bt.45371 | parvin, beta | PARVB | 616294 | 0.6094 | 0.0465 |
| Bt_11759_1_A1_at | Bt.11759 | paired box 6 | PAX6 | 286857 | -0.7176 | 0.0129 |
| Bt_15711_1_S1_a_at | Bt.49118 | poly(rC) binding protein 2 | PCBP2 | 540653 | 0.6302 | 0.0377 |
| Bt_7587_1_S1_a_at | Bt.7587 | poly(rC) binding protein 4 | PCBP4 | 506889 | -0.6410 | 0.0336 |
| Bt_21255_1_A1_a_at | Bt.52040 | polycomb group ring finger 1 | PCGF1 | 539040 | -0.7616 | 0.0065 |
| Bt_13263_1_S1_at | Bt.7113 | pericentriolar material 1 | PCM1 | 525337 | -0.7259 | 0.0114 |
| Bt_22615_1_S1_at | Bt.22615 | Protein-L-isoaspartate (D-aspartate) O-methyltransferase | PCMT1 | 613854 | -0.7176 | 0.0129 |
| Bt_5420_3_A1_at | Bt.5420 | Purkinje cell protein 4 like 1 | PCP4L1 | 100126444 | -0.7524 | 0.0075 |
| Bt_28362_1_S1_at | Bt.18958 | PCTAIRE protein kinase 1 | PCTK1 | 613810 | -0.6622 | 0.0264 |
| Bt_4718_1_S1_at | Bt.4718 | phosphatidylcholine transfer protein | PCTP | 317656 | -0.6858 | 0.0198 |
| Bt_12783_1_S1_at | Bt.12783 | phosducin | PDC | 287007 | -0.7822 | 0.0044 |
| Bt_28023_1_S1_at | Bt.28023 | seminal vesicle secretory protein 109 | PDC-109 | 407187 | -0.6577 | 0.0278 |
| Bt_21191_2_A1_at | Bt.91198 | phosphodiesterase 4D interacting protein | PDE4DIP | 508547 | 0.6228 | 0.0407 |
| Bt_444_1_S1_at | Bt.444 | phosphodiesterase 6C, cGMP-specific, cone, alpha prime | PDE6C | 281975 | -0.6206 | 0.0416 |
| Bt_22306_1_S1_at | Bt.52184 | prenyl (decaprenyl) diphosphate synthase, subunit 1 | PDSS1 | 509696 | -0.7175 | 0.0129 |
| Bt_24903_1_A1_at | Bt.24903 | peroxisomal biogenesis factor 11A | PEX11A | 515608 | -0.7300 | 0.0108 |
| Bt_10901_1_A1_at | Bt.10901 | peroxisomal biogenesis factor 5 | PEX5 | 514832 | -0.6810 | 0.0210 |
| Bt_14675_1_S1_at | Bt.14675 | progastricsin (pepsinogen C) | PGC | 514502 | -0.7875 | 0.0040 |
| Bt_20328_2_S1_a_at | Bt.20328 | PHD finger protein 1 | PHF1 | 508957 | 0.7570 | 0.0070 |
| Bt_19663_1_S1_at | Bt.43054 | PHD finger protein 11 | PHF11 | 513331 | 0.8200 | 0.0020 |
| Bt_10212_1_S1_at | Bt.56381 | Pleckstrin homology-like domain, family A, member 1 | PHLDA1 | 540135 | 0.6806 | 0.0212 |
| Bt_4695_1_S1_at | Bt.4695 | polymeric immunoglobulin receptor | PIGR | 281401 | 0.6775 | 0.0220 |
| Bt_21215_2_S1_at | Bt.18496 | paired-like homeodomain 2 | PITX2 | 506721 | -0.6674 | 0.0249 |
| Bt_1942_1_S1_at | Bt.1942 | plasminogen activator, urokinase receptor | PLAUR | 281983 | 0.6174 | 0.0430 |
| Bt_17280_1_S1_at | --- | similar to Pleckstrin homology domain-containing family H member 1 | PLEKHH1 | 515305 | -0.6603 | 0.0270 |
| Bt_17513_1_A1_at | Bt.17513 | perilipin 5 | PLIN5 | 531199 | -0.6114 | 0.0456 |
| Bt_7584_1_S1_at | Bt.7584 | paraneoplastic antigen MA1 | PNMA1 | 538718 | -0.6914 | 0.0185 |
| Bt_14013_2_S1_at | Bt.14013 | polymerase (RNA) III (DNA directed) polypeptide C (62kD) | POLR3C | 507314 | -0.7559 | 0.0071 |
| Bt_4713_1_S1_at | Bt.8797 | proopiomelanocortin | POMC | 281416 | -0.6855 | 0.0199 |
| Bt_2928_3_S1_a_at | Bt.44514 | protein phosphatase 1, regulatory (inhibitor) subunit 13 like | PPP1R13L | 505818 | -0.6624 | 0.0264 |
| Bt_24282_3_S1_at | Bt.81128 | protein phosphatase 1, regulatory (inhibitor) subunit 15B | PPP1R15B | 538754 | 0.6581 | 0.0277 |
| Bt_22576_3_S1_at | --- | protein phosphatase 1, regulatory (inhibitor) subunit 3D | PPP1R3D | 782160 | 0.6089 | 0.0468 |
| Bt_21482_3_A1_at | Bt.96934 | PR domain containing 1, with ZNF domain | PRDM1 | 538384 | 0.6337 | 0.0363 |
| Bt_4317_2_A1_at | Bt.13142 | protein kinase C, delta | PRKCD | 505708 | -0.6099 | 0.0463 |
| Bt_6669_1_A1_at | Bt.13570 | protein kinase, interferon-inducible double stranded RNA dependent activator | PRKRA | 282875 | -0.6327 | 0.0367 |
| Bt_7197_2_A1_at | Bt.7197 | prolactin | PRL | 280901 | -0.6800 | 0.0213 |
| Bt_12039_1_S1_at | Bt.59472 | protein arginine methyltransferase 2 | PRMT2 | 507839 | -0.6403 | 0.0338 |
| Bt_23633_2_A1_at | Bt.88725 | proline synthetase co-transcribed homolog (bacterial) | PROSC | 509643 | -0.7428 | 0.0088 |
| Bt_29681_1_S1_at | Bt.17 | prolactin-related protein VI | PRP6 | 404081 | -0.7105 | 0.0143 |
| Bt_9982_1_S1_at | Bt.34383 | proline rich 3 | PRR3 | 525344 | -0.6638 | 0.0259 |
| Bt_17831_1_A1_at | Bt.48158 | proline rich Gla (G-carboxyglutamic acid) 4 (transmembrane) | PRRG4 | 616767 | -0.6054 | 0.0484 |
| Bt_22718_1_A1_at | Bt.59585 | proteasome (prosome, macropain) activator subunit 4 | PSME4 | 528142 | -0.7263 | 0.0114 |
| Bt_14010_1_S1_at | Bt.51592 | prostaglandin reductase 1 | PTGR1 | 513177 | -0.6355 | 0.0356 |
| Bt_95_1_S1_at | Bt.95 | parathyroid hormone | PTH | 280903 | -0.6494 | 0.0306 |
| Bt_28518_1_S1_at | Bt.102246 | pancreatic trypsin inhibitor | PTI | 404172 | -0.7494 | 0.0079 |
| Bt_24224_1_S1_at | Bt.24224 | Protein tyrosine phosphatase-like (proline instead of catalytic arginine), member b | PTPLB | 613886 | -0.8138 | 0.0023 |
| Bt_20261_1_S1_at | Bt.53903 | protein tyrosine phosphatase, non-receptor type 3 | PTPN3 | 511010 | -0.6752 | 0.0226 |
| Bt_24848_1_A1_at | Bt.36672 | protein tyrosine phosphatase, receptor type, D | PTPRD | 532751 | -0.6904 | 0.0187 |
| Bt_10398_1_S1_at | Bt.7541 | pentraxin-related gene, rapidly induced by IL-1 beta | PTX3 | 541148 | 0.6022 | 0.0500 |
| Bt_7818_1_S1_at | Bt.7818 | pseudouridylate synthase 1 | PUS1 | 515808 | -0.6105 | 0.0461 |
| Bt_15666_2_S1_at | Bt.48768 | peroxisomal membrane protein 2, 22kDa | PXMP2 | 508609 | -0.6340 | 0.0362 |
| Bt_412_1_S1_at | Bt.412 | glutaminyl-peptide cyclotransferase | QPCT | 281437 | -0.6259 | 0.0394 |
| Bt_15805_1_S1_at | Bt.61431 | R3H domain containing 2 | R3HDM2 | 613499 | -0.6106 | 0.0460 |
| Bt_6140_1_S1_a_at | Bt.6140 | RAB11 family interacting protein 5 (class I) | RAB11FIP5 | 535992 | -0.7363 | 0.0098 |
| Bt_25492_1_A1_at | Bt.25492 | RAB40B, member RAS oncogene family | RAB40B | 534237 | -0.6718 | 0.0236 |
| Bt_4691_1_S1_at | Bt.24043 | RAB5A, member RAS oncogene family | RAB5A | 539764 | 0.6149 | 0.0441 |
| Bt_5491_2_A1_at | Bt.11880 | Rab acceptor 1 (prenylated) | RABAC1 | 512653 | 0.6739 | 0.0230 |
| Bt_10734_1_A1_at | Bt.50127 | RAD21-like 1 (S. pombe) | RAD21L1 | 512408 | -0.7964 | 0.0034 |
| Bt_14078_1_A1_at | Bt.61375 | RAD51 associated protein 1 | RAD51AP1 | 615937 | 0.7884 | 0.0039 |
| Bt_4968_1_S1_at | Bt.4968 | v-raf-1 murine leukemia viral oncogene homolog 1 | RAF1 | 521196 | 0.6467 | 0.0315 |
| Bt_8189_1_A1_at | Bt.8189 | RAN binding protein 1 | RANBP1 | 533251 | -0.6088 | 0.0468 |
| Bt_29754_1_S1_at | --- | RAN binding protein 2 | RANBP2 | 785768 | 0.6643 | 0.0258 |
| Bt_10622_1_S2_at | Bt.10622 | RAP1A, member of RAS oncogene family | RAP1A | 282031 | 0.6471 | 0.0314 |
| Bt_24401_1_A1_at | Bt.17143 | Rap guanine nucleotide exchange factor (GEF) 2 | RAPGEF2 | 100139484 | -0.6590 | 0.0274 |
| Bt_22038_1_S1_a_at | Bt.22038 | arginyl-tRNA synthetase | RARS | 506305 | -0.6164 | 0.0434 |
| Bt_16473_1_A1_at | Bt.57426 | Ras interacting protein 1 | RASIP1 | 505541 | -0.6997 | 0.0165 |
| Bt_13335_1_S1_at | --- | ribokinase | RBKS | 513276 | -0.8618 | 0.0006 |
| Bt_6377_1_S1_at | Bt.49502 | regulator of calcineurin 1 | RCAN1 | 539640 | 0.6649 | 0.0256 |
| Bt_26143_1_A1_at | Bt.43721 | REST corepressor 3 | RCOR3 | 532635 | -0.6713 | 0.0237 |
| Bt_20711_1_S1_at | Bt.20711 | Retinol dehydrogenase 16 (all-trans) | RDH16 | 519940 | -0.6279 | 0.0386 |
| Bt_266_1_S1_at | Bt.266 | retinol dehydrogenase 8 (all-trans) | RDH8 | 281449 | 0.6973 | 0.0171 |
| Bt_12716_1_S1_at | Bt.51680 | REV1 homolog (S. cerevisiae) | REV1 | 513657 | -0.6520 | 0.0297 |
| Bt_28587_1_S1_at | --- | REX4, RNA exonuclease 4 homolog (S. cerevisiae) | REXO4 | 100140713 | -0.6319 | 0.0370 |
| Bt_6837_2_S1_at | Bt.64563 | ring finger and FYVE-like domain containing 1 | RFFL | 530263 | -0.6567 | 0.0282 |
| Bt_4053_1_S1_at | Bt.9918 | regulatory factor X, 2 (influences HLA class II expression) | RFX2 | 534475 | 0.7040 | 0.0156 |
| Bt_4310_1_A1_at | Bt.4310 | regulator of G-protein signaling 1 | RGS1 | 540836 | 0.6869 | 0.0196 |
| Bt_26948_2_S1_at | Bt.97062 | Regulator of G-protein signaling 9 | RGS9 | 281453 | 0.8307 | 0.0015 |
| Bt_17543_1_A1_at | Bt.17543 | hypothetical LOC509065 | RGSL1 | 509065 | -0.6179 | 0.0427 |
| Bt_19348_1_A1_at | Bt.19348 | RIO kinase 2 (yeast) | RIOK2 | 540772 | -0.6927 | 0.0181 |
| Bt_2534_2_S1_at | Bt.76944 | Ribosomal protein L24-like | RLP24 | 783948 | 0.6050 | 0.0486 |
| Bt_16231_1_S1_at | Bt.52934 | required for meiotic nuclear division 1 homolog (S. cerevisiae) | RMND1 | 534105 | -0.6543 | 0.0290 |
| Bt_22955_1_S1_at | Bt.22955 | ribonuclease, RNase A family, 1 (pancreatic) | RNASE1 | 280930 | -0.6270 | 0.0389 |
| Bt_9525_1_A1_at | Bt.9525 | ring finger protein 113A | RNF113A | 493640 | -0.6801 | 0.0213 |
| Bt_6434_2_S1_at | Bt.76784 | Ring finger protein 149 | RNF149 | 506267 | 0.6504 | 0.0303 |
| Bt_27925_1_A1_at | Bt.27925 | ring finger protein 183 | RNF183 | 539200 | -0.6035 | 0.0493 |
| Bt_5022_1_A1_at | Bt.5022 | Ring finger protein 215 | RNF215 | 530174 | -0.7073 | 0.0149 |
| Bt_6205_1_S1_at | Bt.6205 | RNA polymerase II associated protein 1 | RPAP1 | 531362 | -0.7230 | 0.0119 |
| Bt_24726_1_S1_at | Bt.23981 | ribosomal protein L12 | RPL12 | 404133 | 0.6186 | 0.0425 |
| Bt_24483_1_A1_at | Bt.700 | Regulation of nuclear pre-mRNA domain containing 2 | RPRD2 | 614766 | 0.6991 | 0.0167 |
| Bt_1269_2_A1_at | Bt.11675 | ribosomal protein S12 | RPS12 | 326582 | 0.7283 | 0.0110 |
| Bt_24939_1_S1_at | Bt.88378 | regulatory associated protein of MTOR, complex 1 | RPTOR | 507056 | -0.7678 | 0.0058 |
| Bt_12891_1_S1_at | Bt.12891 | Ras-related associated with diabetes | RRAD | 505165 | 0.6585 | 0.0276 |
| Bt_9939_2_S1_at | Bt.9939 | ribosomal RNA processing 9, small subunit (SSU) processome component, homolog (yeast) | RRP9 | 614025 | -0.6519 | 0.0298 |
| Bt_24622_1_S1_at | Bt.44966 | radial spoke 3 homolog (Chlamydomonas) | RSPH3 | 533135 | -0.6227 | 0.0407 |
| Bt_21401_3_A1_at | Bt.14645 | radial spoke head 9 homolog (Chlamydomonas) | RSPH9 | 523327 | -0.6475 | 0.0313 |
| Bt_2441_1_S1_at | Bt.2441 | rhotekin | RTKN | 539691 | -0.6468 | 0.0315 |
| Bt_21396_1_A1_s_at | Bt.16079 | reticulon 3 | RTN3 | 359721 | -0.6068 | 0.0477 |
| Bt_21301_1_S1_a_at | Bt.29191 | reticulon 4 interacting protein 1 | RTN4IP1 | 506626 | -0.6234 | 0.0405 |
| Bt_357_1_S1_at | Bt.357 | S100 calcium binding protein A12 (calgranulin C) | S100A12 | 282467 | 0.6516 | 0.0299 |
| Bt_9360_1_S1_at | Bt.9360 | S100 calcium binding protein A8 | S100A8 | 616818 | 0.6243 | 0.0401 |
| Bt_278_1_S1_at | Bt.49986 | serum amyloid A 3 | SAA3 | 281474 | 0.6198 | 0.0419 |
| Bt_18085_2_A1_at | Bt.18085 | SREBF chaperone | SCAP | 507878 | 0.8577 | 0.0007 |
| Bt_2867_2_A1_at | Bt.2867 | Schwannomin interacting protein 1 | SCHIP1 | 535716 | -0.6073 | 0.0475 |
| Bt_17845_1_A1_at | Bt.28635 | SEC23 interacting protein | SEC23IP | 508557 | -0.6784 | 0.0217 |
| Bt_22483_1_S1_at | Bt.43173 | SEC31 homolog B (S. cerevisiae) | SEC31B | 507880 | -0.6478 | 0.0311 |
| Bt_23336_1_S1_at | Bt.16905 | Secreted and transmembrane 1 | SECTM1 | 781942 | -0.6283 | 0.0384 |
| Bt_2336_1_S1_at | Bt.56822 | Septin 3 | Sep-03 | 618235 | -0.6224 | 0.0408 |
| Bt_10471_1_A1_at | Bt.72389 | serpin peptidase inhibitor, clade A (alpha-1 antiproteinase, antitrypsin), member 12 | SERPINA12 | 777604 | -0.6427 | 0.0329 |
| Bt_235_1_S1_at | Bt.49228 | serpin peptidase inhibitor, clade A (alpha-1 antiproteinase, antitrypsin), member 7 | SERPINA7 | 282518 | -0.6185 | 0.0425 |
| Bt_29678_1_A1_at | Bt.28437 | serpin peptidase inhibitor, clade B (ovalbumin), member 6 | SERPINB6 | 404051 | -0.6844 | 0.0202 |
| Bt_2638_1_S1_at | Bt.2638 | serpin peptidase inhibitor, clade F (alpha-2 antiplasmin, pigment epithelium derived factor), member 1 | SERPINF1 | 281386 | 0.6086 | 0.0469 |
| Bt_86_1_S1_at | Bt.9352 | serpin peptidase inhibitor, clade F (alpha-2 antiplasmin, pigment epithelium derived factor), member 2 | SERPINF2 | 282522 | 0.6129 | 0.0450 |
| Bt_22969_1_S1_at | Bt.22969 | serpin peptidase inhibitor, clade G (C1 inhibitor), member 1 | SERPING1 | 281035 | 0.6301 | 0.0377 |
| Bt_11057_1_S1_at | Bt.43083 | shisa homolog 3 (Xenopus laevis) | SHISA3 | 781091 | -0.7582 | 0.0068 |
| Bt_9195_1_A1_at | Bt.9195 | SIVA1, apoptosis-inducing factor | SIVA1 | 617931 | -0.6192 | 0.0422 |
| Bt_21213_1_S1_at | Bt.21213 | SKI-like oncogene | SKIL | 527910 | 0.6456 | 0.0319 |
| Bt_16250_2_S1_at | Bt.51814 | solute carrier family 10 (sodium/bile acid cotransporter family), member 1 | SLC10A1 | 532890 | -0.8753 | 0.0004 |
| Bt_8328_1_S1_at | Bt.46957 | solute carrier family 11 (proton-coupled divalent metal ion transporters), member 2 | SLC11A2 | 521189 | -0.6114 | 0.0457 |
| Bt_16486_2_A1_at | Bt.52086 | solute carrier family 13 (sodium/sulfate symporters), member 4 | SLC13A4 | 531535 | -0.7300 | 0.0108 |
| Bt_29906_1_S1_at | Bt.29906 | solute carrier family 22 (organic anion transporter), member 7 | SLC22A7 | 407224 | -0.7322 | 0.0104 |
| Bt_26918_1_S1_at | Bt.26918 | solute carrier family 29 (nucleoside transporters), member 2 | SLC29A2 | 531564 | -0.7276 | 0.0111 |
| Bt_6359_1_S1_at | Bt.6359 | solute carrier family 30 (zinc transporter), member 4 | SLC30A4 | 540869 | -0.6108 | 0.0459 |
| Bt_8828_1_S1_at | Bt.46260 | Solute carrier family 38, member 10 | SLC38A10 | 509536 | 0.7167 | 0.0131 |
| Bt_22998_1_S1_at | Bt.29369 | solute carrier family 5 (sodium-dependent vitamin transporter), member 6 | SLC5A6 | 516021 | -0.6989 | 0.0167 |
| Bt_23_1_S1_at | Bt.23 | solute carrier family 6 (neurotransmitter transporter, noradrenalin), member 2 | SLC6A2 | 282363 | -0.7140 | 0.0136 |
| Bt_4627_1_S2_at | Bt.88817 | solute carrier family 6 (neurotransmitter transporter, creatine), member 8 | SLC6A8 | 282367 | -0.6454 | 0.0320 |
| Bt_5290_1_S1_at | Bt.5290 | structural maintenance of chromosomes 3 | SMC3 | 281729 | -0.6277 | 0.0387 |
| Bt_7562_1_S1_at | Bt.7562 | sphingomyelin phosphodiesterase 4, neutral membrane (neutral sphingomyelinase-3) | SMPD4 | 507207 | -0.7679 | 0.0058 |
| Bt_29074_1_A1_at | Bt.32813 | SET and MYND domain containing 3 | SMYD3 | 616050 | -0.7336 | 0.0102 |
| Bt_251_1_S1_at | Bt.251 | synuclein, gamma (breast cancer-specific protein 1) | SNCG | 281494 | -0.6255 | 0.0396 |
| Bt_13962_1_S1_at | Bt.13962 | sorting nexin 15 | SNX15 | 507751 | -0.6375 | 0.0349 |
| Bt_1736_1_A1_at | Bt.1736 | suppressor of cytokine signaling 1 | SOCS1 | 518795 | 0.6904 | 0.0187 |
| Bt_16110_1_A1_at | Bt.101558 | SON DNA binding protein | SON | 516462 | -0.6248 | 0.0399 |
| Bt_19638_1_S1_at | Bt.41234 | sperm autoantigenic protein 17 | SPA17 | 616974 | -0.6151 | 0.0440 |
| Bt_21619_1_S1_at | Bt.21619 | spastic paraplegia 20 (Troyer syndrome) | SPG20 | 534027 | -0.6870 | 0.0195 |
| Bt_9636_1_S1_at | Bt.9636 | serine peptidase inhibitor, Kazal type 1 | SPINK1 | 574092 | 0.6221 | 0.0410 |
| Bt_508_1_A1_at | Bt.508 | secreted phosphoprotein 2, 24kDa | SPP2 | 281500 | -0.6067 | 0.0478 |
| Bt_5951_1_S1_at | Bt.45761 | splA/ryanodine receptor domain and SOCS box containing 4 | SPSB4 | 512922 | -0.7032 | 0.0158 |
| Bt_2219_1_S1_at | Bt.47373 | signal recognition particle 14kDa (homologous Alu RNA binding protein) | SRP14 | 512792 | 0.6408 | 0.0336 |
| Bt_10332_1_A1_at | Bt.22353 | serine/arginine repetitive matrix 2 | SRRM2 | 539515 | -0.8696 | 0.0005 |
| Bt_25416_1_A1_at | Bt.25416 | Slingshot homolog 1 (Drosophila) | SSH1 | 538233 | -0.6726 | 0.0234 |
| Bt_10272_1_S1_at | Bt.10272 | stanniocalcin 1 | STC1 | 338078 | 0.6446 | 0.0323 |
| Bt_3704_3_A1_at | Bt.3704 | stathmin-like 2 | STMN2 | 534991 | -0.7216 | 0.0122 |
| Bt_27449_1_A1_at | Bt.91956 | suppressor of fused homolog (Drosophila) | SUFU | 535067 | -0.6180 | 0.0427 |
| Bt_774_1_S1_at | Bt.15527 | surfeit 6 | SURF6 | 511610 | -0.6435 | 0.0327 |
| Bt_27168_1_A1_a_at | Bt.51444 | TAO kinase 3 | TAOK3 | 534620 | 0.6185 | 0.0425 |
| Bt_27935_1_A1_at | Bt.101041 | TAR DNA binding protein | TARDBP | 540632 | -0.7016 | 0.0161 |
| Bt_27808_1_S1_at | Bt.81172 | TatD DNase domain containing 3 | TATDN3 | 615965 | -0.6315 | 0.0372 |
| Bt_3308_1_S1_at | Bt.97014 | TBC1 domain family, member 24 | TBC1D24 | 529002 | -0.6314 | 0.0372 |
| Bt_3535_1_S1_at | Bt.57675 | TBC1 domain family, member 9B (with GRAM domain) | TBC1D9B | 514521 | -0.6086 | 0.0469 |
| Bt_9385_1_S1_at | Bt.49077 | tubulin folding cofactor C | TBCC | 515354 | 0.6548 | 0.0288 |
| Bt_26770_1_S1_at | Bt.26770 | transducin (beta)-like 2 | TBL2 | 511758 | 0.6459 | 0.0318 |
| Bt_21870_1_S1_at | Bt.59682 | transcription factor 25 (basic helix-loop-helix) | TCF25 | 618386 | 0.6393 | 0.0342 |
| Bt_8976_1_S1_at | Bt.43658 | Transcription factor 7-like 2 (T-cell specific, HMG-box) | TCF7L2 | 540481 | -0.6141 | 0.0444 |
| Bt_16091_1_S1_at | Bt.43244 | Trichoplein, keratin filament binding | TCHP | 522380 | -0.6625 | 0.0263 |
| Bt_22336_1_A1_at | Bt.22336 | teratocarcinoma-derived growth factor 1 | TDGF1 | 784029 | -0.7116 | 0.0141 |
| Bt_13745_2_S1_at | Bt.13745 | tudor domain containing 3 | TDRD3 | 537918 | -0.6325 | 0.0368 |
| Bt_11257_1_S1_at | Bt.11257 | Telomeric repeat binding factor 2 | TERF2 | 506501 | -0.6744 | 0.0229 |
| Bt_11240_1_A1_at | --- | tet oncogene family member 2 | TET2 | 538481 | -0.6927 | 0.0181 |
| Bt_16460_1_A1_at | Bt.65767 | Testis expressed 9 | TEX9 | 518432 | -0.6418 | 0.0333 |
| Bt_19112_1_A1_at | Bt.39597 | TRK-fused gene | TFG | 505171 | -0.7165 | 0.0131 |
| Bt_24864_2_A1_at | Bt.24864 | tissue factor pathway inhibitor (lipoprotein-associated coagulation inhibitor) | TFPI | 508763 | 0.6810 | 0.0210 |
| Bt_11260_2_S1_at | Bt.74006 | trans-golgi network protein 2 | TGOLN2 | 511904 | 0.6047 | 0.0488 |
| Bt_23611_3_S1_at | Bt.23611 | TCDD-inducible poly(ADP-ribose) polymerase | TIPARP | 540975 | 0.6253 | 0.0397 |
| Bt_29710_1_A1_at | Bt.59823 | tight junction protein 3 (zona occludens 3) | TJP3 | 407100 | 0.6442 | 0.0324 |
| Bt_9567_1_S1_at | Bt.9567 | transmembrane 7 superfamily member 2 | TM7SF2 | 282384 | -0.6706 | 0.0239 |
| Bt_27845_2_S1_at | Bt.39532 | Transmembrane and coiled-coil domain family 1 | TMCC1 | 510486 | -0.6367 | 0.0352 |
| Bt_8094_1_S1_at | Bt.8094 | transmembrane emp24 protein transport domain containing 9 | TMED9 | 618580 | 0.6581 | 0.0277 |
| Bt_8848_1_S1_at | Bt.8848 | transmembrane protein 106C | TMEM106C | 514271 | -0.6595 | 0.0273 |
| Bt_25421_1_S1_at | Bt.37966 | transmembrane protein 161A | TMEM161A | 618720 | -0.6551 | 0.0287 |
| Bt_13294_1_A1_at | Bt.13294 | transmembrane protein 161B | TMEM161B | 510378 | -0.6040 | 0.0491 |
| Bt_9850_2_A1_at | Bt.9850 | Transmembrane protein 171 | TMEM171 | 538802 | -0.7869 | 0.0041 |
| Bt_15565_1_S1_at | Bt.53871 | transmembrane protein 176A | TMEM176A | 404082 | 0.7540 | 0.0073 |
| Bt_21250_1_S1_a_at | Bt.21250 | transmembrane protein 176B | TMEM176B | 615505 | 0.7229 | 0.0120 |
| Bt_14260_1_A1_at | Bt.14260 | transmembrane protein 177 | TMEM177 | 505501 | -0.6066 | 0.0478 |
| Bt_20497_2_S1_a_at | Bt.76856 | transmembrane protein 179B | TMEM179B | 512135 | 0.6192 | 0.0422 |
| Bt_8147_1_S1_at | Bt.45994 | transmembrane protein 187 | TMEM187 | 508380 | 0.6401 | 0.0339 |
| Bt_15859_1_S1_at | Bt.15859 | transmembrane protein 214 | TMEM214 | 514683 | 0.6313 | 0.0372 |
| Bt_28251_1_S1_at | Bt.28251 | transmembrane protein 220 | TMEM220 | 504970 | 0.6025 | 0.0498 |
| Bt_28540_1_A1_at | Bt.28540 | transmembrane protein 44 | TMEM44 | 614057 | -0.6147 | 0.0442 |
| Bt_27323_1_S1_at | Bt.27323 | Transmembrane protein 52 | TMEM52 | 617403 | -0.6106 | 0.0460 |
| Bt_21761_1_S1_a_at | Bt.87251 | tropomodulin 4 (muscle) | TMOD4 | 505645 | -0.8254 | 0.0018 |
| Bt_29035_1_A1_at | Bt.29035 | tumor necrosis factor, alpha-induced protein 1 (endothelial) | TNFAIP1 | 539674 | 0.6619 | 0.0265 |
| Bt_27752_1_S1_at | Bt.28890 | tensin 4 | TNS4 | 532898 | 0.7064 | 0.0151 |
| Bt_1563_1_S1_at | Bt.1563 | transducer of ERBB2, 2 | TOB2 | 507934 | -0.7611 | 0.0065 |
| Bt_21839_1_A1_at | Bt.39255 | Topoisomerase (DNA) I | TOP1 | 534799 | 0.6916 | 0.0184 |
| Bt_4964_1_S1_at | Bt.91168 | tumor protein p53 binding protein 1 | TP53BP1 | 509111 | -0.6169 | 0.0432 |
| Bt_26672_1_S1_at | Bt.16627 | tumor protein p53 inducible nuclear protein 1 | TP53INP1 | 782667 | 0.7731 | 0.0053 |
| Bt_17716_1_A1_at | Bt.28741 | TPX2, microtubule-associated, homolog (Xenopus laevis) | TPX2 | 507226 | -0.6555 | 0.0285 |
| Bt_13003_14_A1_at | --- | T cell receptor, alpha | TRA@ | 511486 | -0.7524 | 0.0075 |
| Bt_25049_1_S1_at | --- | translocation associated membrane protein 1-like 1 | TRAM1L1 | 505068 | -0.7576 | 0.0069 |
| Bt_1978_10_A1_a_at | --- | T cell receptor, beta cluster | TRB@ | 281547 | 0.7820 | 0.0045 |
| Bt_17761_1_A1_at | Bt.17761 | tripartite motif-containing 68 | TRIM68 | 538657 | -0.6249 | 0.0398 |
| Bt_19959_2_A1_at | Bt.19959 | Transient receptor potential cation channel, subfamily A, member 1 | TRPA1 | 505317 | 0.6330 | 0.0366 |
| Bt_22515_1_S1_a_at | Bt.22515 | transient receptor potential cation channel, subfamily V, member 2 | TRPV2 | 507664 | 0.6533 | 0.0293 |
| Bt_19750_1_A1_at | Bt.19750 | TRNA selenocysteine associated protein 1 | TRSPAP1 | 532582 | -0.6187 | 0.0424 |
| Bt_20335_1_A1_at | Bt.20335 | TruB pseudouridine (psi) synthase homolog 2 (E. coli) | TRUB2 | 511988 | -0.7552 | 0.0072 |
| Bt_5839_1_S1_at | --- | tRNA splicing endonuclease 2 homolog (S. cerevisiae) | TSEN2 | 515511 | -0.6407 | 0.0337 |
| Bt_5393_1_S1_at | Bt.39887 | tetratricopeptide repeat domain 3 | TTC3 | 506569 | -0.6922 | 0.0183 |
| Bt_22419_1_A1_at | --- | tubulin, gamma complex associated protein 5 | TUBGCP5 | 512912 | -0.7181 | 0.0128 |
| Bt_25753_1_A1_at | Bt.64594 | thioredoxin domain containing 6 | TXNDC6 | 513348 | -0.6822 | 0.0207 |
| Bt_17929_1_A1_at | Bt.17929 | Ubiquitin-like modifier activating enzyme 5 | UBA5 | 509292 | 0.7001 | 0.0164 |
| Bt_2174_1_S1_at | Bt.37042 | ubiquitin-like modifier activating enzyme 6 | UBA6 | 518834 | -0.6453 | 0.0320 |
| Bt_20602_1_S1_at | Bt.55450 | ubiquitin-conjugating enzyme E2B (RAD6 homolog) | UBE2B | 512207 | 0.6604 | 0.0270 |
| Bt_6350_1_A1_at | Bt.4543 | ubiquitin-conjugating enzyme E2H (UBC8 homolog, yeast) | UBE2H | 539313 | 0.6765 | 0.0223 |
| Bt_24878_1_A1_at | Bt.24878 | ubiquitin-conjugating enzyme E2O | UBE2O | 789422 | -0.7120 | 0.0140 |
| Bt_2915_1_S1_at | Bt.2915 | ubiquitin-conjugating enzyme E2T (putative) | UBE2T | 505314 | -0.6289 | 0.0382 |
| Bt_12245_1_S1_at | Bt.14654 | ubiquitin protein ligase E3C | UBE3C | 508513 | 0.6829 | 0.0206 |
| Bt_4658_1_S1_a_at | Bt.88820 | ubiquitin-like 4A | UBL4A | 504533 | -0.6423 | 0.0331 |
| Bt_1089_1_A1_at | Bt.1089 | ubiquitin-like domain containing CTD phosphatase 1 | UBLCP1 | 508163 | -0.6223 | 0.0409 |
| Bt_4772_2_S1_a_at | Bt.43985 | Ubiquilin 4 | UBQLN4 | 533021 | -0.6835 | 0.0204 |
| Bt_21551_2_S1_a_at | Bt.21551 | uridine-cytidine kinase 1-like 1 | UCKL1 | 534046 | -0.6340 | 0.0362 |
| Bt_110_1_S1_at | Bt.110 | uroplakin 2 | UPK2 | 281569 | 0.6276 | 0.0387 |
| Bt_18788_1_S1_at | Bt.49828 | Uroporphyrinogen decarboxylase | UROD | 504914 | -0.7810 | 0.0045 |
| Bt_13886_1_S1_at | Bt.102041 | Ubiquitin specific peptidase 34 | USP34 | 534067 | 0.6525 | 0.0295 |
| Bt_13342_1_S1_at | Bt.13342 | UTP15, U3 small nucleolar ribonucleoprotein, homolog (S. cerevisiae) | UTP15 | 526343 | -0.6392 | 0.0342 |
| Bt_4138_2_S1_at | Bt.4138 | vascular endothelial growth factor A | VEGFA | 281572 | -0.7173 | 0.0130 |
| Bt_29875_1_S1_at | Bt.5484 | very low density lipoprotein receptor | vldlr | 282123 | -0.7888 | 0.0039 |
| Bt_27865_1_S1_at | Bt.27865 | Vpr (HIV-1) binding protein | VPRBP | 541079 | 0.6036 | 0.0493 |
| Bt_13592_1_A1_at | Bt.28232 | vacuolar protein sorting 24 homolog (S. cerevisiae) | VPS24 | 507722 | -0.6596 | 0.0272 |
| Bt_17824_3_S1_at | Bt.17824 | vacuolar protein sorting 36 homolog (S. cerevisiae) | VPS36 | 511223 | -0.6471 | 0.0314 |
| Bt_26765_1_S1_at | Bt.26765 | vacuolar protein sorting 8 homolog (S. cerevisiae) | VPS8 | 615672 | -0.7862 | 0.0041 |
| Bt_28764_1_A1_at | Bt.17255 | vaccinia related kinase 3 | VRK3 | 520302 | -0.7966 | 0.0033 |
| Bt_21128_1_S1_at | Bt.21128 | Vps20-associated 1 homolog (S. cerevisiae) | VTA1 | 506430 | -0.6991 | 0.0167 |
| Bt_10781_2_S1_at | --- | WD repeat domain 17 | WDR17 | 783416 | -0.6044 | 0.0489 |
| Bt_8290_1_S1_at | Bt.8290 | WD repeat, sterile alpha motif and U-box domain containing 1 | WDSUB1 | 783784 | -0.6883 | 0.0192 |
| Bt_21004_1_S1_at | Bt.34215 | WAP four-disulfide core domain 3 | WFDC3 | 505523 | -0.6447 | 0.0322 |
| Bt_15927_2_S1_at | Bt.15927 | WD repeat domain, phosphoinositide interacting 1 | WIPI1 | 528410 | 0.7300 | 0.0108 |
| Bt_27254_2_S1_at | Bt.27254 | wingless-type MMTV integration site family, member 2B | WNT2B | 445420 | -0.7372 | 0.0096 |
| Bt_20351_1_S1_at | Bt.20351 | WD repeat containing, antisense to TP53 | WRAP53 | 509631 | -0.6471 | 0.0314 |
| Bt_14579_1_A1_at | Bt.66386 | Werner helicase interacting protein 1 | WRNIP1 | 782711 /// 788824 | -0.7053 | 0.0153 |
| Bt_5403_1_S1_at | Bt.5403 | xanthine dehydrogenase | XDH | 280960 | 0.6105 | 0.0461 |
| Bt_11847_1_A1_at | --- | X (inactive)-specific transcript | XIST | 338325 | 0.8643 | 0.0006 |
| Bt_12054_1_S1_at | Bt.12054 | Yip1 domain family, member 1 | YIPF1 | 511748 | -0.6248 | 0.0399 |
| Bt_27340_1_A1_at | --- | YLP motif containing 1 | YLPM1 | 540849 | -0.7542 | 0.0073 |
| Bt_19375_1_S1_at | Bt.19375 | YTH domain family, member 1 | YTHDF1 | 538525 | -0.7267 | 0.0113 |
| Bt_20159_1_S1_at | Bt.20159 | zinc finger CCCH-type containing 18 | ZC3H18 | 523958 | -0.7156 | 0.0133 |
| Bt_29758_1_A1_at | Bt.4674 | zinc finger, C3HC-type containing 1 | ZC3HC1 | 533550 | -0.8504 | 0.0009 |
| Bt_20849_1_S1_at | Bt.42317 | zinc finger, CCHC domain containing 11 | ZCCHC11 | 528986 | -0.6190 | 0.0423 |
| Bt_17584_1_S1_at | Bt.20533 | zinc finger, AN1-type domain 6 | ZFAND6 | 514167 | -0.6735 | 0.0231 |
| Bt_25120_1_S1_at | Bt.25120 | zinc finger RNA binding protein | ZFR | 538494 | -0.7697 | 0.0056 |
| Bt_24685_1_A1_at | Bt.24685 | Zinc finger, FYVE domain containing 26 | ZFYVE26 | 514402 | -0.6295 | 0.0379 |
| Bt_10218_1_S1_s_at | Bt.20278 | zinc finger, MYM-type 3 | ZMYM3 | 522721 | -0.6073 | 0.0475 |
| Bt_27422_1_A1_at | Bt.42658 | zinc finger, MYND-type containing 12 | ZMYND12 | 512257 | -0.6092 | 0.0466 |
| Bt_17848_2_S1_at | Bt.52435 | zinc finger, MYND-type containing 8 | ZMYND8 | 506031 | -0.6359 | 0.0355 |
| Bt_17868_1_A1_at | Bt.17868 | zinc finger protein 317 | ZNF317 | 506859 | -0.6999 | 0.0165 |
| Bt_1136_1_S1_at | Bt.76288 | zinc finger protein 329 | ZNF329 | 100138653 | -0.6026 | 0.0497 |
| Bt_17985_1_S1_at | Bt.17985 | zinc finger protein 362 | ZNF362 | 508065 | -0.6788 | 0.0216 |
| Bt_8699_1_S1_at | Bt.8699 | zinc finger protein 385A | ZNF385A | 540045 | -0.6065 | 0.0479 |
| Bt_21907_1_S1_at | Bt.21907 | zinc finger protein 48 | ZNF48 | 512477 | -0.6522 | 0.0296 |
| Bt_11027_1_A1_at | Bt.11027 | zinc finger protein 653 | ZNF653 | 516232 | -0.7735 | 0.0052 |
| Bt_6874_1_S1_at | Bt.6874 | zinc finger protein 786 | ZNF786 | 506408 | -0.6228 | 0.0407 |
| Bt_28670_1_A1_at | Bt.79609 | zyg-11 homolog B (C. elegans) | ZYG11B | 540744 | 0.7523 | 0.0076 |
| Bt_17105_1_A1_at | Bt.27487 | zinc finger, ZZ-type containing 3 | ZZZ3 | 538498 | 0.6787 | 0.0217 |
